# Whole-genome sequencing of *Puccinia striiformis* f. sp. *tritici* mutant isolates identifies avirulence gene candidates

**DOI:** 10.1186/s12864-020-6677-y

**Published:** 2020-03-20

**Authors:** Yuxiang Li, Chongjing Xia, Meinan Wang, Chuntao Yin, Xianming Chen

**Affiliations:** 1 Department of Plant Pathology, Washington State University, Pullman, WA 99164-6430 USA; 2 USDA-ARS, Wheat Health, Genetics, and Quality Research Unit, Pullman, WA 99164-6430 USA

**Keywords:** Stripe rust, *Puccinia striiformis*, Avirulence, Effector, Genomics, Mutation, Wheat, Yellow rust

## Abstract

**Background:**

The stripe rust pathogen, *Puccinia striiformis* f. sp. *tritici* (*Pst*), threats world wheat production. Resistance to *Pst* is often overcome by pathogen virulence changes, but the mechanisms of variation are not clearly understood. To determine the role of mutation in *Pst* virulence changes, in previous studies 30 mutant isolates were developed from a least virulent isolate using ethyl methanesulfonate (EMS) mutagenesis and phenotyped for virulence changes. The progenitor isolate was sequenced, assembled and annotated for establishing a high-quality reference genome. In the present study, the 30 mutant isolates were sequenced and compared to the wide-type isolate to determine the genomic variation and identify candidates for avirulence (*Avr*) genes.

**Results:**

The sequence reads of the 30 mutant isolates were mapped to the wild-type reference genome to identify genomic changes. After selecting EMS preferred mutations, 264,630 and 118,913 single nucleotide polymorphism (SNP) sites and 89,078 and 72,513 Indels (Insertion/deletion) were detected among the 30 mutant isolates compared to the primary scaffolds and haplotigs of the wild-type isolate, respectively. Deleterious variants including SNPs and Indels occurred in 1866 genes. Genome wide association analysis identified 754 genes associated with avirulence phenotypes. A total of 62 genes were found significantly associated to 16 avirulence genes after selection through six criteria for putative effectors and degree of association, including 48 genes encoding secreted proteins (SPs) and 14 non-SP genes but with high levels of association (*P* ≤ 0.001) to avirulence phenotypes. Eight of the SP genes were identified as avirulence-associated effectors with high-confidence as they met five or six criteria used to determine effectors.

**Conclusions:**

Genome sequence comparison of the mutant isolates with the progenitor isolate unraveled a large number of mutation sites along the genome and identified high-confidence effector genes as candidates for avirulence genes in *Pst.* Since the avirulence gene candidates were identified from associated SNPs and Indels caused by artificial mutagenesis, these avirulence gene candidates are valuable resources for elucidating the mechanisms of the pathogen pathogenicity, and will be studied to determine their functions in the interactions between the wheat host and the *Pst* pathogen.

## Background

*Puccinia striiformis* f. sp. *tritici* (*Pst*), the causal agent of wheat stripe (yellow) rust, is a threat to wheat production worldwide [1]. Wheat stripe rust can cause 100% yield loss on susceptible cultivars in a single field when weather conditions are favorable for infection, but generally can cause up to 10% yield losses in large-scale regions or countries [1]. In the global scale, billions of dollars are spent annually on fungicide application for reducing stripe rust damage. Growing resistant cultivars is an effective and environmentally friendly way to control stripe rust. However, resistant cultivars may become susceptible few years after releasing due to virulence changes in the pathogen population [2, 3]. For example, the breakdown of *Yr17* by *Pst* virulence races led to the epidemics of stripe rust in northern Europe from 1993 to 1999 [4]. In the recent decades, the *Pst* virulence spectrum has become wider and the *Pst* population is getting more aggressive in Europe, North America and other continents [2, 3, 5–7]. Taking the US as an example, the total identified races and emerging races are much higher in 2000–2009 than in 1968–1999 [6]. Accordingly, gaining a better understanding of mechanisms of *Pst* variation is crucial for monitoring *Pst* populations and developing strategies for more efficient control of stripe rust.

Mutation and somatic and sexual recombination have been demonstrated as principal mechanisms causing *Pst* variation [8–10]. Mutation is proposed to be the most important approach in creating new *Pst* races and genotypes [10]. Considering efficiency and power to produce mutations, ethyl methanesulfonate (EMS) is the most popular mutagen used by researchers in studying mutants of various organisms. EMS is an alkylating agent, which is known as inducing base substitutions in the genome strands. Of the single nucleotide polymorphisms (SNPs) caused by EMS, C/G to T/A transitions were most frequent in various organisms, including *Arabidopsis thaliana* [11, 12], *Caenorhabditis elegans* [13, 14], *Lotus japonicus* [15], *Oryza sativa* [12, 16] and *Saccharomyces cerevisiae* [17]. In addition to point mutations, EMS is able to generate insertions and deletions in genome sequences, which may result in phenotypic changes as well [11, 13, 18, 19]. In rust fungi, Li et al. [10] developed a *Pst* mutant population through EMS mutagenesis and characterized the population with virulence and molecular markers. Salcedo et al. [20] obtained EMS-induced urediniospore mutants from the wheat stem rust pathogen *Puccinia graminis* f. sp. *tritici* (*Pgt*) Ug99, which led to the cloning of avirulence (*Avr*) gene *AvrSr35*. Mutagenesis integrated with genomic sequencing is an efficient way to study the relationships between phenotypic traits and associated genes, leading to the identification of fungal effectors or avirulence genes. The similar strategy has also been applied in cloning resistance genes in plant hosts [21].

Identifying and cloning avirulence genes are based on the gene-for-gene hypothesis proposed by Flor [22], which states that host *R* genes confer resistance to the cognate *Avr* genes in the pathogen. During the infection of pathogens, the first layer of host defense is pathogen-associated molecular pattern (PAMP)-triggered immunity (PTI). When PTI is crashed by pathogen effectors, stronger defense responses, referred as effector-triggered immunity (ETI), are triggered, leading to hypersensitive responses [23]. The increasing variation of pathogen virulence is due to the arm race between pathogen Avr effectors and corresponding host resistance (R) proteins, causing the rapid evolution of the pathogen [24]. To date, a handful of *Avr* genes have been molecularly identified in rust pathogens, including *AvrL567*, *AvrP123*, *AvrP4*, *AvrM*, *AvrL2* and *AvrM14* from the flax rust pathogen *Melampsora lini* (*M. lini*) together with *PGTAUSPE10–1*, *AvrSr35* and *AvrSr50* from *Pgt* [18, 25–28]. In *Pst*, Dagvadorj et al. [29] reported that *PstSCR1* can activate immunity in non-host plants. Zhao et al. [30] found that *Pst_8713* was involved in enhancing *Pst* virulence and suppressing plant immunity. Yang et al. [31] identified that *Pst18363* displayed an important pathogenicity factor in *Pst.* However, no known *Avr* genes have been identified in *Pst* so far.

With the rapid development of sequencing technologies, the genome sequences of *Pst* are available, which makes it possible to further understand the pathogenesis of the obligate biotrophic fungal parasite [32–38]. The advancement of genome sequencing has led ever-expanding candidate effector genes identified in *Pst*. Cantu et al. [33] identified five *Pst* candidate effector genes from 2999 predicted secreted protein (SP) genes. Xia et al. [39] predicted a set of 25 *Pst Avr* candidate genes from 2146 predicted SPs by combining comparative genomics with association analyses. Similar approaches were also used in detecting *Avr* candidate genes in *Puccinia triticina* (*Pt*), the wheat leaf rust pathogen [40]. These predicted effectors are determined based on the characteristics from previous identified effectors. In rust pathogens, even with some exceptions, most effectors have shared some common features, such as secreted, small size, cysteine-rich, species-specific, polymorphic, no conserved protein domains and haustorially expressed [33, 41–43]. Unlike a conserved motif RxLR noted in oomycetes effectors [44], no common sequence motifs of fungi effectors were detected through bioinformatic analyses [45]. One of the sporadic exceptions is in the barley powdery mildew pathogen, *Blumeria graminis* f. sp. *hordei* (*Bgh*) with some effectors sharing a conserved N-terminal [Y/F/W]xC motif [46]. This motif has also been reported in rust fungi, *Melampsora larici*-*populina* and *Pgt*, but not limited to the N-terminal region [47]. Even though there is no a one-size-fits-all standard to identify candidate effectors, those features are still useful in detecting effectors in an expanding number of fungal species.

To determine and characterize the potential Avr effectors in *Pst*, in the present study we generated and analyzed whole-genome sequences of EMS-induced mutants. By comparing with the progenitor isolate genome, SNPs and Indels (Insertion/deletion) were found from the mutant isolates. By filtering out the low-quality and low-impact variants, genome association analyses identified 754 genes significantly associated with *Pst* avirulence/virulence phenotypes. We further predicted 48 genes as SP genes and 8 of them as putative effector genes with high confidence. Additionally, fourteen non-SP genes that were highly associated (*P* ≤ 0.001) to individual avirulence genes were also worthy being studied for their effects on avirulence. This study was the first in *Pst* that integrated mutagenesis, genomics analysis and association analysis for mining effectors. The identified avirulence candidates should be further studied to determine their functions in the plant-pathogen interactions, providing useful information for developing new approaches for monitoring the pathogen population and more effective strategies for controlling the disease.

## Results

### Virulence characterization of the progenitor and mutant isolates

**Fig. 1:**
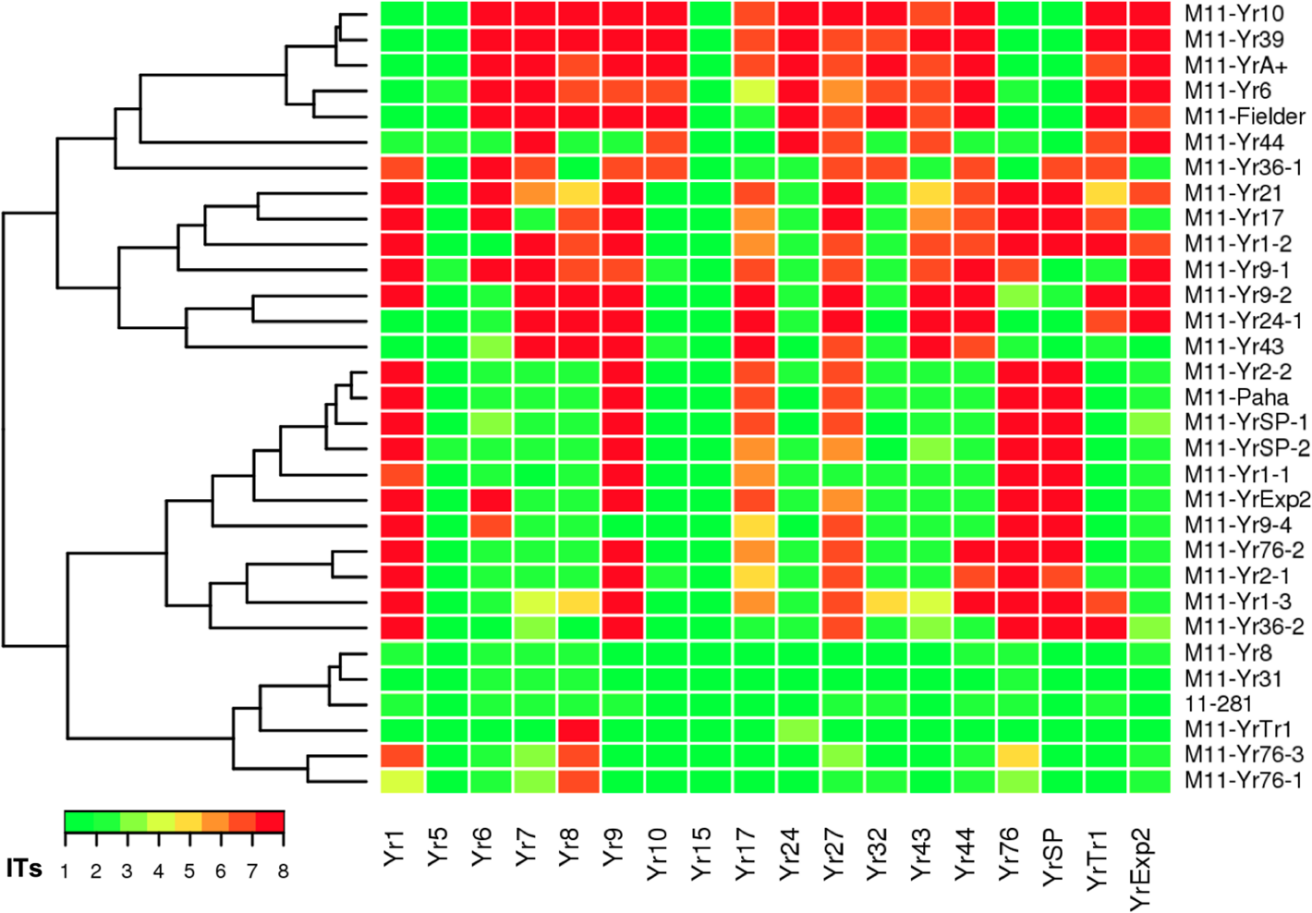
Heatmap and dendrogram of wild-type isolate 11–281 and its mutants of *Puccinia striiformis* f. sp. *tritici* based on infection types (ITs). The virulence characterization of all isolates was conducted on the 18 wheat *Yr* single-gene differentials [48]. ITs 1 to 8 were transformed to the color key ranging from green to red, which indicate avirulent (resistant) to virulent (susceptible) reactions

The *Pst* isolate 11–281 was chosen as the progenitor isolate because it is avirulent on all 18 *Yr* single-gene lines used to differentiate *Pst* races. Thirty mutant isolates were selected for the present study from 33 EMS-induced mutant isolates based on their avirulence/virulence patterns characterized on the 18 wheat *Yr* single-gene differentials in the previous study [10]. Compared with the infection types (IT 1 or 2) of the wild-type isolate on the 18 wheat differentials, changes from avirulence to virulence occurred on all *Yr* single-gene lines to different extents except for *Yr5* and *Yr15*. Thus, phenotypic changes of avirulence to virulence could be studied for avirulence genes corresponding to 16 *Yr* resistance genes (*Yr1*, *Yr6*, *Yr7*, *Yr8*, *Yr9*, *Yr10*, *Yr17*, *Yr24*, *Yr27*, *Yr32*, *Yr43*, *Yr44*, *YrSP*, *YrTr1*, *YrExp2* and *Yr76*) using the 30 selected mutant isolates. The IT data of the wild-type isolate and 30 mutant isolates and the frequency of virulent mutant isolates are provided in Additional file 1: Table S1, and the IT patterns of the 30 mutant isolates on the 18 *Yr* single-gene lines, as well as a dendrogram showing their relationships based on the IT data, are illustrated in Fig. 1. The frequencies of the changed virulence factors corresponding to the 16 *Yr* genes among the 30 mutant isolates ranged from 21.2% (*Yr32*) to 78.8% (*Yr9*). The relative balances of avirulent to virulent phenotypes among the 30 mutant isolates indicate that these isolates are suitable for studying markers related to the 16 avirulence/virulence loci using associate analysis.

### Genome alignment and sequence variation

The high-quality genome (accession SBIN00000000) of the progenitor isolate (11–281), obtained through PacBio, Illumina and RNA sequencing as previously reported [49], was used as the reference genome in the present study. The assembled sequence comprised 381 primary scaffolds and 873 haplotigs with the genome size of 84.75 Mb and 60.09 Mb, 16,869 and 12,145 protein-coding genes and 1829 and 1318 SP genes, respectively. The mutant isolates were sequenced by Illumina sequencing with an estimated average coverage of 30x. The 30 raw reads are publicly available in the National Center of Biological Information (NCBI) with SRA accession SRR10413520 to SRR10413549. After aligning the 30 mutant sequences to the reference genome and treating the alignment by a series of analytical software, BAM files were obtained. The mapping rates of alignments ranged from 65.36 to 71.93% by comparing with the primary scaffolds and 56.36 to 62.50% with the haplotigs of the wild-type isolate genome (Additional file 1: Table S2).

**Fig. 2:**
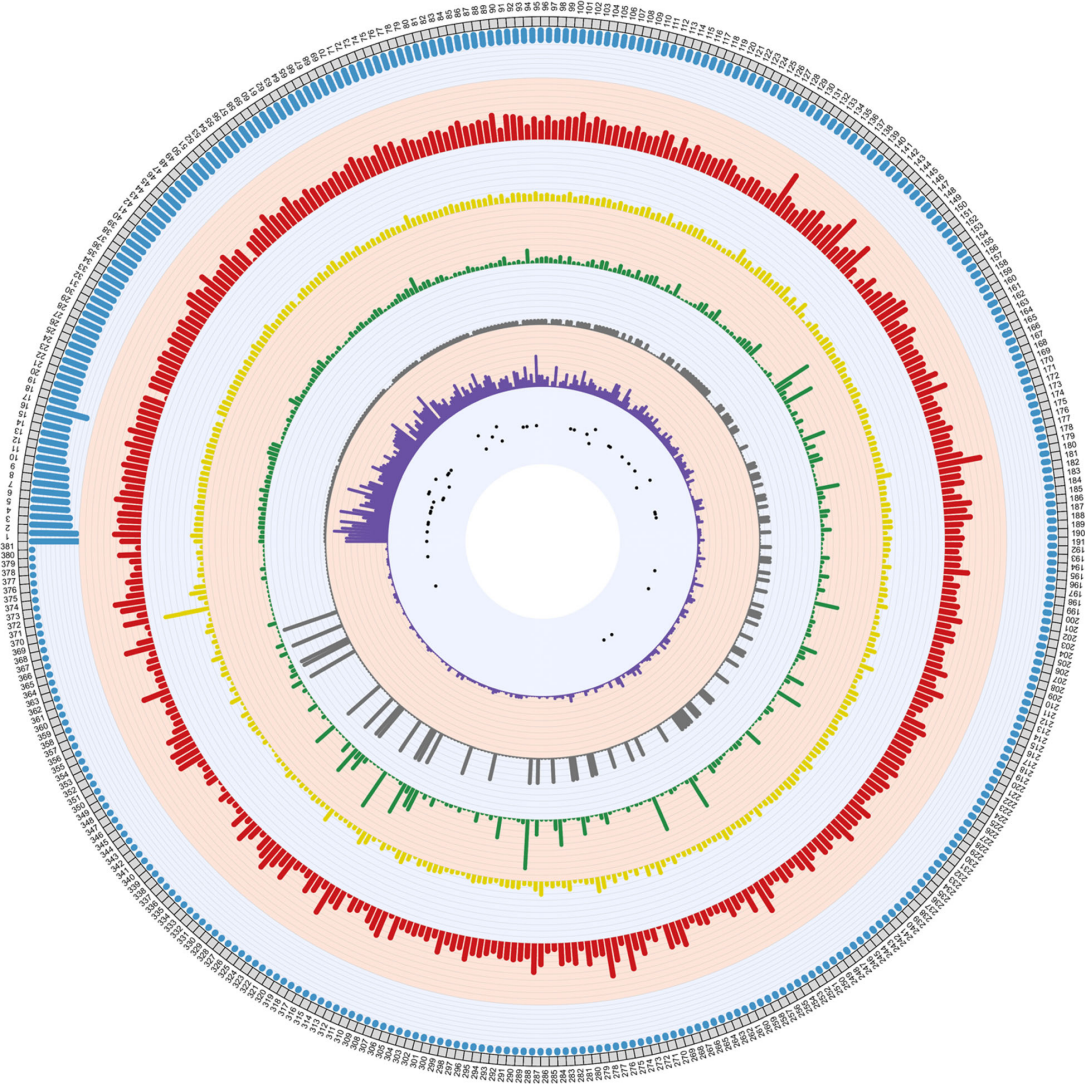
Genome-wide identification of variants (SNPs and/or Indels), variants densities, distribution of secreted proteins (SPs) and effector candidates from primary assembled scaffolds. The grey bars in the outer layer are the scaffolds of the reference genome, and each axis indicates the genome size of 150 Kb. The first layer in red and second layer in yellow indicate SNP and Indel densities throughout the genome, respectively. Each axis represents 1000 SNPs or Indels per Mb. The third layer in green and the fourth layer in grey exhibits densities if deleterious SNPs and Indel in the scaffolds. Each axis shows 70 SNPs or Indels per Mb. The fifth layer in purple displayed the distribution of SPs in the genome, and each axis indicates 4 SPs. The black dots in the inner layer represent the effector candidates distributed in the scaffolds

By mapping the Illumina reads of the wild-type isolates (11–281) to the reference genomes, we identified 196,350 SNPs and 173,075 Indels from its primary scaffolds and 48,647 SNPs and 7612 Indels from its haplotigs. The heterozygous sites were then removed from the variants we obtained. After separating variants from the alignments and keeping only the EMS-induced SNPs, the number of SNPs ranged from 9353 to 117,035 among the 30 mutants detected from the primary scaffolds. The heterozygous rates extended from 70.92 to 99.07% (Table 1). The densities and distribution of SNPs on the primary scaffolds were displayed in Fig. 2 and Additional file 1: Table S3. A phylogenetic tree was constructed to show the genetic relationships among the mutant isolates using the SNPs (Additional file 2: Fig. S1), indicating that EMS mutagenesis is able to create various degrees of genomic variation. The number of Indels ranged from 4005 to 20,705 in the 30 mutant isolates. The most frequent Indel length was 1 bp (46.90%), followed by 2 bp (18.69%) and 3 bp (8.65%), counting for 74.24% Indels. To the extreme, a 273-bp insertion and a 245-bp deletion were the largest Indels detected in this study (Additional file 1: Table S4). Likewise, the Indel distribution and density varied among scaffolds (Fig. 2; Additional file 1: Table S3). SNPs and Indels were also identified from the haplotigs, and the results were displayed in Additional file 1: Table S5.

**Table 1:** Numbers and percentages of heterozygous and homozygous of EMS-induced SNPs in mutant isolates of *Puccinia striiformis* f. sp. *tritici* detected by mapping to the primary scaffolds of isolate 11–281

Mutant	No. of SNPs	Heterozygous		Homozygous	
isolates		No.	Percent (%)^a^	No.	Percent (%)^b^
M11-Yr36–1	117,035	109,070	91.84	3726	8.16
M11-Yr21	115,281	103,417	71.05	17,707	28.95
M11-YrTr1	113,504	99,896	97.78	208	2.22
M11-Yr24–1	112,757	98,611	93.91	5463	6.09
M11-Yr9–2	109,247	100,435	97.94	201	2.06
M11-Yr17	100,118	92,493	99.07	227	0.93
M11-Yr39	89,676	84,213	71.65	17,350	28.35
M11-Yr10	86,237	80,478	93.54	5508	6.46
M11-YrA+	86,114	80,365	97.74	223	2.26
M11-Yr9–1	85,723	80,208	93.57	5515	6.43
M11-Fielder	85,258	79,750	87.45	14,146	12.55
M11-Yr6	85,195	79,658	88.01	13,608	11.99
M11-Yr9–4	72,356	61,995	71.52	17,402	28.48
M11-Yr1–2	71,929	60,913	92.49	3470	7.51
M11-Yr1–3	62,064	53,728	93.32	5749	6.68
M11-YrSP-1	61,507	44,014	91.93	8812	8.07
M11-Yr2–1	61,352	43,571	98.12	191	1.88
M11-Yr76–2	61,243	43,742	71.02	17,781	28.98
M11-YrExp2	61,198	43,848	70.92	17,722	29.08
M11-Yr2–2	61,171	43,464	85.68	10,361	14.32
M11-Paha	61,094	43,692	86.57	8336	13.43
M11-Yr36–2	60,944	43,222	93.32	5759	6.68
M11-YrSP-2	60,943	43,717	71.42	17,501	28.58
M11-Yr76–1	46,186	42,716	89.71	11,864	10.29
M11-Yr76–3	45,671	41,945	93.19	7965	6.81
M11-Yr43	24,453	24,226	92.38	7625	7.62
M11-Yr44	10,139	9948	71.56	17,493	28.44
M11-Yr1–1	9881	9658	71.73	17,226	28.27
M11-Yr8	9768	9567	84.68	11,016	15.32
M11-Yr31	9353	9145	93.50	5537	6.50
**Average**	**67,913**	**58,724**	**86.47**	**9190**	**13.53**

^a^The percentage of heterozygous SNPs was calculated as the number of heterozygous SNPs divided by the total number of SNPs of each isolate times 100

^b^The percentage of heterozygous SNPs was calculated as the number of homozygous SNPs divided by the total number of SNPs of each isolate times 100

**Fig. 3:**
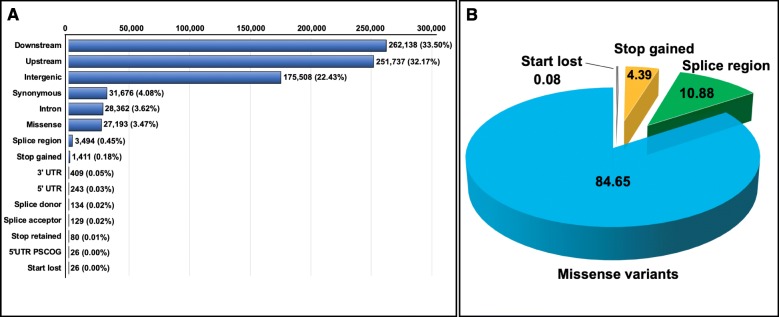
Types and frequencies of SNP effects detected from the primary scaffolds. **a**: The number and percentage of all EMS-induced SNPs for each type of effects. 5′ UTR PSCOG is the acronym of 5′ UTR premature start codon gain variant. Stop gained and start lost indicted the variants derived from gaining a stop codon and losing a start codon. **b**: The types and percentages of SNP effects, including missense, splice, stop gained and start lost variants, were identified as deleterious effects, and the number of each SNP effect indicates the percentage contributed to the total deleterious effects

**Fig. 4:**
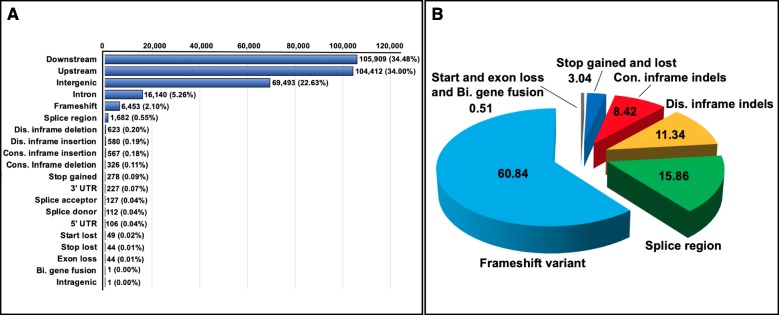
Types and frequencies of Indels effects found from the primary scaffolds. **a**: The number and percentage of all EMS-induced Indels for each type of effects. Bi. is the abbreviation of bidirectional gene fusion, indicates fusion of two genes in opposite directions. Con. and Dis. are the abbreviation of conserved and disruptive. Splice acceptor and donor mean the variant hits a splice acceptor site. **b**: The types and percentages of deleterious Indels effects. The number shows the proportion in percentage of each variant effect out of the total deleterious effects

Prediction of the effects of the variants on the genome was implemented using SnpEff. It should be noted that one variant might cause multiple effects in the genome, the 264,630 and 118,913 SNPs derived from the primary scaffolds and haplotigs accounted for 782,566 and 369,000 effects, respectively. The 89,078 and 72,513 Indels caused 307,134 and 250,464 effects, respectively. The effect types of SNPs and frequency of each category were displayed in Fig. 3a. The effects of SNPs were mainly in downstream (33.50%), upstream (32.17%) and intergenic (22.43%) regions, followed by synonymous (4.08%), intron (3.62%) and missense (3.47%) variants. Since missense, splice, start loss and stop gain variants were predicted to have a moderate or high impact on the genome, those variants were regarded as deleterious mutations resulting in the impact on gene functions (http://snpeff.sourceforge.net/SnpEff_manual.html). Missense variants were the predominant (84.65%) among all the deleterious mutations (Fig. 3b). Similarly, the effects of Indels were mostly in downstream (34.48%), upstream (34.00%) and intergenic (22.63%) regions (Fig. 4a) The percentage of moderate to high-impact effect were illustrated in Fig. 4b, of which frameshift variants were the most frequent (60.84%) among all deleterious Indels. The types and frequencies of SNP and Indel effects detected from the haplotigs are displayed in Additional file 2: Fig. S2 and Fig. S3.

### *Pst* effector genes as candidates for *Avr* genes

Deleterious SNPs and Indels were selected from the associated variants according to their impact on the genome. Deleterious variants detected from the primary scaffolds and haplotigs were analysed and summarized in Table 2 and Table 3, respectively. As shown in Table 2, deleterious SNPs extended from 133 (M11-Yr8 and M11-Yr31) to 1821 (M11-Yr36–1) with the involving genes ranging from 66 (M11-Yr8) to 682 (M11-Yr36–1). Of the deleterious Indels, the number ranged from 40 (M11-Yr8) to 271 (M11-YrTr1) involving in 28 (M11-Yr8) to 125 (M11-YrTr1) genes. These SNPs and Indels were found to be involved in 1135 genes (Table 2). Similarly, deleterious variants identified from the haplotigs varied among different mutant isolates with 731 involving genes (Table 3). Overall, 1866 genes were inferred from the variants detected from both the primary scaffolds and haplotigs.

**Table 2:** Numbers of deleterious SNPs, Indels and corresponded genes in mutant isolates of *Puccinia striiformis* f. sp. *tritici* detected from the primary scaffolds of isolate 11–281

Mutant Isolate	Deleterious SNPs^a^	Deleterious SNPs on genes^b^	Deleterious Indels^c^	Deleterious indels on genes	Deleterious SNPs and Indels on genes
M11-Yr36–1	1821	682	217	121	716
M11-Yr21	1820	664	254	123	698
M11-YrTr1	1612	641	271	125	682
M11-Yr24–1	1722	650	201	106	678
M11-Yr9–2	1695	651	218	117	688
M11-Yr17	1528	609	164	92	640
M11-Yr39	1371	544	120	77	582
M11-Yr10	1280	492	120	85	522
M11-YrA+	1273	509	128	76	537
M11-Yr9–1	1295	501	144	74	529
M11-Fielder	1317	503	176	91	532
M11-Yr6	1280	499	170	95	532
M11-Yr9–4	1134	491	152	98	543
M11-Yr1–2	1132	505	173	109	566
M11-Yr1–3	972	426	163	101	482
M11-YrSP-1	988	425	192	115	486
M11-Yr2–1	981	426	202	114	491
M11-Yr76–2	970	423	198	114	488
M11-YrExp2	965	424	190	120	492
M11-Yr2–2	971	421	187	111	484
M11-Paha	943	416	189	113	480
M11-Yr36–2	952	419	216	121	490
M11-YrSP-2	960	421	171	105	483
M11-Yr76–1	844	323	112	69	357
M11-Yr76–3	838	318	109	64	349
M11-Yr43	391	217	93	56	247
M11-Yr44	142	81	78	36	100
M11-Yr1–1	151	77	58	31	99
M11-Yr8	133	66	40	28	83
M11-Yr31	133	72	54	32	88
**Total**^**d**^	**4217**	**1042**	**1122**	**412**	**1135**

^a^Associated deleterious SNPs were selected based on the types of variants annotated using the SnpEff program. SNPs with moderate and high effects were considered as deleterious SNPs

^b^Associated genes were deduced from the annotated file generated using the SnpEff program. Multiple SNPs can occur in one gene

^c^Associated deleterious indels were selected based on the types of variants annotated using the SnpEff program. Indels with moderate and high effects were considered as deleterious Indels

^d^SNPs, Indels and genes can be shared in different mutant isolates, so the total number is not equal to summation of individuals

**Table 3:** Numbers of deleterious SNPs, indels and corresponded genes in mutant isolates of *Puccinia striiformis* f. sp. *tritici* detected from the haplotigs of isolate 11–281

Mutant Isolate	Deleterious SNPs	Deleterious SNPs on genes	Deleterious Indels	Deleterious Indels on genes	Deleterious variants on genes
M11-Yr36–1	147	85	73	50	124
M11-Yr21	243	125	146	95	192
M11-YrTr1	211	127	158	92	190
M11-Yr24–1	476	215	349	175	326
M11-Yr9–2	226	119	123	69	170
M11-Yr17	578	278	250	140	360
M11-Yr39	348	174	227	117	252
M11-Yr10	248	135	165	96	202
M11-YrA+	347	166	217	112	242
M11-Yr9–1	218	116	133	78	177
M11-Fielder	233	116	148	95	183
M11-Yr6	180	99	123	74	154
M11-Yr9–4	664	304	383	207	417
M11-Yr1–2	806	338	557	252	476
M11-Yr1–3	334	184	161	111	259
M11-YrSP-1	393	213	261	156	309
M11-Yr2–1	777	354	545	264	491
M11-Yr76–2	461	235	316	185	352
M11-YrExp2	392	218	269	157	317
M11-Yr2–2	844	363	631	280	522
M11-Paha	714	329	500	244	462
M11-Yr36–2	757	338	560	265	493
M11-YrSP-2	340	181	228	129	266
M11-Yr76–1	140	78	70	44	110
M11-Yr76–3	243	125	144	83	189
M11-Yr43	89	59	6	6	65
M11-Yr44	76	46	9	9	49
M11-Yr1–1	118	60	10	10	67
M11-Yr8	55	30	9	9	38
M11-Yr31	69	37	7	7	41
**Total**	**1895**	**564**	**1059**	**401**	**731**

To identify inferred genes associated to avirulence, genome-wide association analysis was conducted using the avirulence/virulence phenotype data and genes with deleterious mutants. Genes with probability (*P*) values ≤0.05 in the association analysis were regarded as significantly associated with the avirulence/virulence phenotypes. Predicted effector candidates were obtained from the associated proteins based on the criteria of with N-terminal signal peptide and without transmembrane helix. A total of 754 genes were found significantly associated with 16 *Avr* loci, of which 48 SP genes were predicted to be effector candidate genes (Table 4, Table 5). Associated SP genes were identified for all 16 *Avr* loci that had varied phenotypes among the 30 mutant isolates. *AvYr27* had the highest number of associated genes (149). *AvYr27* also had the most associated SP genes (10) together with *AvYr7*. *AvYr8* had 27 associated genes including 1 SP gene. Only one SP gene was found for each of *AvYr1*, *AvYr24* and *AvYr76* (Table 4).

**Table 4:** Numbers of associated genes, associated genes with signal peptides, SP genes without transmembrane helices (TH), and genes highly associated to avirulence (*Avr*) genes

*Avr*	No. of	No. of associated genes	No. of associated	No. of highly
gene	associated genes^a^	with signal peptides^b^	SP genes^c^	associated genes^d^
*AvYr1*	62	2	1	0
*AvYr6*	129	9	9	1
*AvYr7*	121	11	10	1
*AvYr8*	27	1	1	4
*AvYr9*	83	3	2	2
*AvYr10*	70	3	3	0
*AvYr17*	54	3	3	0
*AvYr24*	35	1	1	1
*AvYr27*	149	11	10	4
*AvYr32*	79	4	4	1
*AvYr43*	100	7	6	0
*AvYr44*	58	6	5	0
*AvYr76*	48	1	1	0
*AvYrExp2*	76	4	3	0
*AvYrSP*	117	7	7	4
*AvYrTr1*	45	6	6	0
**Total**^**e**^	**754**	**54**	**48**	**17**

^a^Associated genes to each *Avr* gene were selected with the *P* value ≤0.05 of output files using the GAPIT program

^b^Signal peptides were detected from associated genes using SignalP 5.0 program

^c^Proteins of associated genes with signal peptides and without transmembrane helices were considered as SP genes

^d^Associated genes to *Avr* genes with the *P* value ≤0.001 were considered as highly associated genes. Highly associated genes include 14 Non-SP genes and 3 SP genes

^e^One gene can be associated with multiple *Avr* genes, so the total number of genes is not equal to the summation of associated genes from each *Avr* gene

**Table 5:** Characterization of 48 *Avr* effector candidates of *Puccinia striiformis* f. sp. *tritici* derived from secreted protein genes

*Avr*	Associated	SPL^b^	EffectorP		AA^d^	C^e^		Polymorphic^f^	Pfam	Motif^g^
candidate	*Avr* gene^a^	%	Effector	%^c^	length	%	Conservation	in *Pst*	Family	
PS_11–281_00004726	1	99.8	Yes	67.4	115	5.22	*Ps.* specific	Yes		[Y/F/W]xC
PS_11–281_00016865	6	99.8	Yes	88.5	115	6.09	*P*^h^*.* specific	Yes		[Y/F/W]xC
PS_11–281_00015631	7	99.7	Yes	94.5	133	6.02	*P.* specific	Yes		
PS_11–281_00002472	7	99.8	Yes	84.7	153	3.92	*P.* specific	Yes		RXLR-Like; [Y/F/W]xC
PS_11–281_00009923	76	99.9	Yes	85.2	127	5.51	*Ps.* specific	Yes		[Y/F/W]xC
PS_11–281_haploid_00011016	17	99.7	Yes	94.5	133	6.02	*P.* specific	Yes		[Y/F/W]xC
PS_11–281_00011501	32, SP	67.7	Yes	57.4	307	4.56	*Ps.* specific	Yes		[Y/F/W]xC
PS_11–281_00002262	8	96.3	No	73.3	112	4.46	*Ps.* specific	Yes		RXLR-Like; [Y/F/W]xC
PS_11–281_00010750	27	97.5	No	88.1	506	3.56	*P.* specific	Yes		
PS_11–281_00012402	7, 27	95.7	No	51	140	0.00	*Ps.* specific	Yes		
PS_11–281_00014479	7, 27, 43, Exp2	83.1	No	95.7	186	3.23	Basidiomycetes orthologs	Yes		
PS_11–281_haploid_00010301	6	97.5	No	95.7	524	3.82	*P.* specific	Yes		
PS_11–281_00009924	Tr1	81.5	No	95.7	1371	0.58	*P.* specific	Yes		
PS_11–281_00009841	44	98.8	No	91.5	783	0.13	*P.* specific	Yes		
PS_11–281_00009866	17	60.9	No	88.8	406	1.23	*Ps.* specific	Yes		
PS_11–281_00013192	27	97.1	No	92.1	627	0.64	*Ps.* specific	Yes		
PS_11–281_00014125	SP	98.2	No	96.9	329	3.65	/^i^	Yes		
PS_11–281_00016661	43, Exp2, SP	92.5	No	96.9	574	0.35	*Ps.* specific	Yes		
PS_11–281_00003286	27	89.2	No	92.6	250	0.80	*P.* specific	Yes		RXLR-Like
PS_11–281_00011083	6, SP	96.3	No	99	936	1.60	*P.* specific	Yes		
PS_11–281_00014412	6, SP	84.6	No	95.6	696	1.58	*P.* specific	Yes		
PS_11–281_haploid_00003208	10, 32	79.8	No	91.2	468	0.21	*P.* specific	Yes		
PS_11–281_haploid_00006920	6	85.7	No	99.1	524	1.15	*P.* specific	Yes		[L/I]xAR
PS_11–281_haploid_00006507	6	99.0	No	95.4	589	0.85	*P.* specific	Yes		RXLR-Like
PS_11–281_haploid_00002163	9, 17	60.9	No	93.4	443	1.81	*Ps.* specific	Yes		
PS_11–281_haploid_00004265	6	57.8	No	94.9	1325	4.53	*Ps.* specific	Yes	PF00840.20	
PS_11–281_haploid_00002648	7, Tr1	66.9	No	97.6	812	0.86	*P.* specific	Yes		
PS_11–281_00004920	7, 27, 43, Tr1	72.2	No	86	323	0.93	/	Yes		RXLR-Like
PS_11–281_00014999	27	96.6	No	93.6	1029	0.29	/	Yes		
PS_11–281_00001788	27	66.8	No	98.9	1989	1.16	*P.* specific	Yes	PF03033.20	RXLR-Like; [Y/F/W]xC
PS_11–281_00011666	10, 24, 27, Tr1	92.0	No	95.1	1165	0.09	*P.* specific	Yes	PF02358.16	
PS_11–281_00011857	SP	99.8	No	98.8	760	1.48	/	Yes		RXLR-Like
PS_11–281_00003160	43	97.5	No	99.1	993	0.91	*P.* specific	Yes	PF00450.22	
PS_11–281_00006608	27	60.2	No	97.3	550	2.00	*P.* specific	Yes	PF01575.19	
PS_11–281_00012767	7, 43, Exp2	92.2	No	94.3	1933	0.16	/	Yes		
PS_11–281_00003315	10, 32	99.7	No	90.5	400	1.25	*P.* specific	Yes	PF02156.15	
PS_11–281_haploid_00005879	44	87.1	No	98.9	670	0.60	/	Yes		
PS_11–281_00011495	6	92.8	No	95.6	1206	1.16	/	Yes	PF01735.18	
PS_11–281_00013988	Tr1	62.3	No	78.9	416	0.48	/	Yes	PF12776.7	
PS_11–281_00003121	7, 43, 44	65.0	No	97.6	1498	0.93	Basidiomycetes orthologs	Yes	PF01239.22	
PS_11–281_00005153	Tr1	79.2	No	93.9	1524	0.46	/	Yes	PF01369.20	RXLR
PS_11–281_00013742	7	97.6	No	94	406	0.74	/	Yes	PF05890.12	
PS_11–281_00012902	7	99.5	No	97.8	507	0.79	/	Yes	PF01565.23	
PS_11–281_00012863	9, SP	55.0	No	88.2	848	1.06	/	Yes	PF07217.11	
PS_11–281_haploid_00007789	6	56.5	No	97.1	649	0.92	/	Yes	PF09118.11	
PS_11–281_haploid_00010769	44	91.0	No	88.1	452	1.33	Basidiomycetes orthologs	Yes	PF00704.28	
PS_11–281_haploid_00005482	32	99.9	No	99	630	0.95	/	Yes	PF09286.11	
PS_11–281_haploid_00011285	44	60.6	No	98.7	541	1.11	/	Yes	PF00135.28	

^a^Associated *Avr* gene included 1, 6, 7, 8, 9,10,17, 24, 27, 32, 43, 44, 76, Exp2, SP and Tr1, which are the abbreviations for the *AvYr1*, *AvYr6*, AvYr7, *AvYr8*, *AvYr9*, *AvYr10*, *AvYr17*, *AvYr24*, *AvYr27*, *AvYr32*, *AvYr43*, *AvYr44*, *AvYr76*, *AvYrExp2*, *AvYrSP* and *AvYrTr1*, respectively

^b^SPL = signal peptide likelihood

^c^ The percentage indicates the probability of the protein to be effector or non-effector based on the machine learning evaluation in EffectorP

^d^*AA* Amino acid

^e^*C* Cysteine

^f^The polymorphisms of effector candidates were determined by searching homologous sequences from publicly available protein database in *Pst*

^g^Motifs RxLR, RxLR-like ([R/K/H]x[L/M/I/F/Y/W]x), YxSL[R/K], [L/I] xAR, [R/K]CxxCx12H, [R/K]VY[L/I] R, G[I/F/Y][A/L/S/T]R and [Y/F/W]xC were searched for motifs in the effector candidates

^h^*P Puccinia*

^i^/ No conservation detected in the Fungi Kingdom

**Fig. 5:**
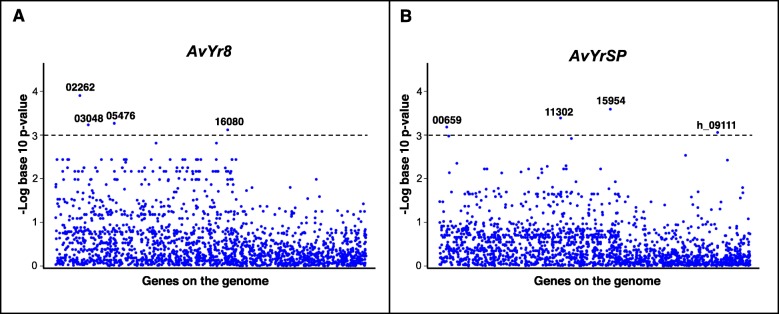
Examples of Manhattan plots in scaffolds displaying association between genes from isolate 11–281 and *Avr* genes of *Puccinia striiformis* f. sp. *tritici*. The X axis shows genes on the genome and Y axis indicates -log_10_ (*P* value). **a**: Genes are highly associated with *AvYr8*. 02262, 03048, 05476 and 16,080 are PS_11–281_00002262, PS_11–281_00003048, PS_11–281_00005476 and PS_11–281_00016080, which are associated to *AvYr8* with *P* value 1.25E-04, 5.83E-04, 5.40E-04 and 7.63E-04, respectively. **b**: Genes are highly associated with *AvYrSP*. 00659, 11,302, 15,954 and h_09111 denote PS_11–281_00000659, PS_11–281_00011302, PS_11–281_00015954 and PS_11–281_haploid_00009111, respectively, which are associated to *AvYrSP* with *P* value 6.53E-04, 4.11E-04, 2.58E-04 and 8.74E-04, respectively

To identify highly associated genes, 17 genes with *P* values ≤0.001 were identified from the 754 associated genes, of which 3 were SP and 14 were non-SP genes. The 17 genes were found highly associated to eight *Avr* loci, including *AvYr6*, *AvYr7*, *AvYr8*, *AvYr9*, *AvYr24, AvYr27, AvYr32* and *AvYrSP*. Four genes were associated to *AvYr8*, *AvYr27* and *AvYrSP*, two genes to *AvYr9* and one gene to *AvYr6*, *AvYr7*, *AvYr24* and *AvYr32*. As an example, four genes associated to *AvYr8* and *AvYrSP* are shown in Fig. 5a and Fig. 5b. Except for one gene (PS_11–281_haploid_00002745), which was associated to *AvYr24* and *AvYr32*, each of the other 16 genes was associated to a single *Avr* locus. Of these 17 highly associated genes, missense variants were the majority. Five genes had frameshifts, 3 had gained stop codons, 2 had Inframe insertions and only 1 lost the start codon (Additional file 1: Table S6). Fourteen out of the 17 highly associated genes were not SP genes (Table 6). Thus, a total of 62 genes, including 48 effector candidate genes and 14 non-SP genes, were considered as candidates for avirulence genes. Their genomic locations and derived amino acids are provided in Additional file 3: Table SE1.

**Table 6:** Characterization of 14 *Avr* effector candidates of *Puccinia striiformis* f. sp. *tritici* from non-secreted protein genes

*Avr*	Associated	SPL	EffectorP		AA	C		Polymorphic	Pfam	Motif
candidate	*Avr* gene	%	Effector	%	length	%	Conservation	in *Pst*	Family	
PS_11–281_00005476	*AvYr8*	0.8	No	50.5	381	3.9	*Ps.* specific	Yes		[Y/F/W]xC
PS_11–281_00009555	*AvYr9*	0.1	No	99.1	590	3.2	*P.* specific	Yes		
PS_11–281_00015954	*AvYrSP*	1.6	No	87.7	283	0.4	*P.* specific	Yes		
PS_11–281_00005142	*AvYr27*	0.4	No	99	902	2.8	*Ps.* specific	Yes		[Y/F/W]xC
PS_11–281_haploid_00011106	*AvYr6*	0.1	No	95.9	564	2.3	/	Yes		
PS_11–281_00014472	*AvYr7*	0.1	No	96.8	612	1.8	/	Yes		[L/I]xAR
PS_11–281_00000659	*AvYrSP*	0.3	No	73.3	287	0.7	/	Yes	PF14223.6	
PS_11–281_00003048	*AvYr8*	0.1	No	95.6	1363	0.4	/	Yes	PF09766.9	
PS_11–281_00016080	*AvYr8*	0.1	No	99.1	1415	1.4	/	Yes	PF07727.14	
PS_11–281_00000903	*AvYr9*	0.5	No	99.1	527	0.8	Basidiomycetes orthologs	Yes	PF00617.19	
PS_11–281_haploid_00002745	*AvYr24*; *AvYr32*	0.1	No	99.1	611	1.6	/	Yes	PF07690.16	
PS_11–281_00003874	*AvYr27*	0.1	No	99.1	1087	2.3	/	Yes	PF00899.21	[Y/F/W]xC
PS_11–281_00011302	*AvYrSP*	0.1	No	97.1	1222	2.1	/	Yes	PF05699.14	[Y/F/W]xC
PS_11–281_haploid_00009111	*AvYrSP*	0.1	No	99.1	823	2.4	/	Yes	PF00648.21	

### Characterization of *Pst* effector gene candidates

**Fig. 6:**
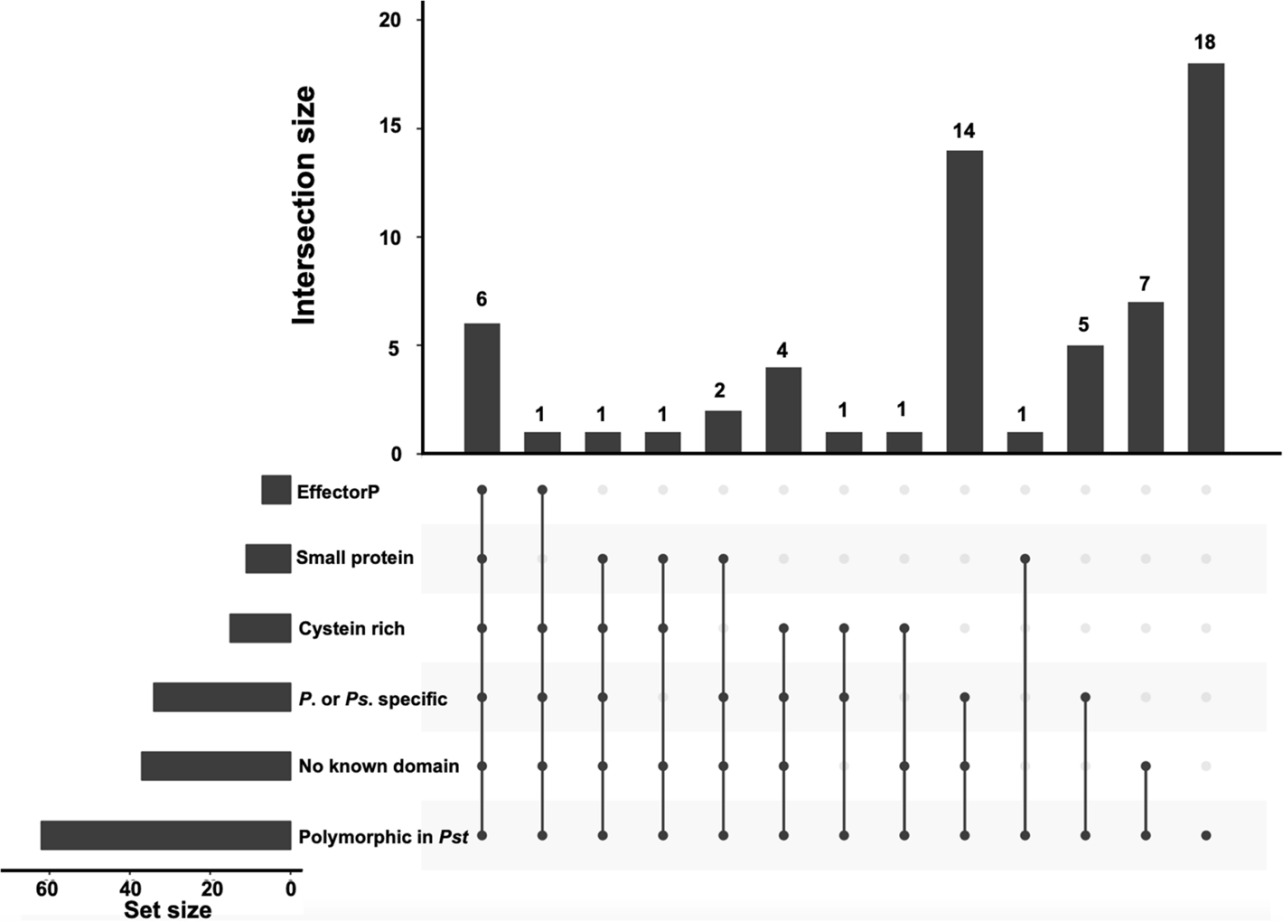
Characterization of 62 *Puccinia striiformis* f. sp. *tritici Avr* effector candidates in Upset plots. Six criteria were used for characterizing the effector candidates. The set size indicates the number of candidates meet the requirement for each criterion. The intersection size shows the number of candidates meeting one or more criteria

**Fig. 7:**
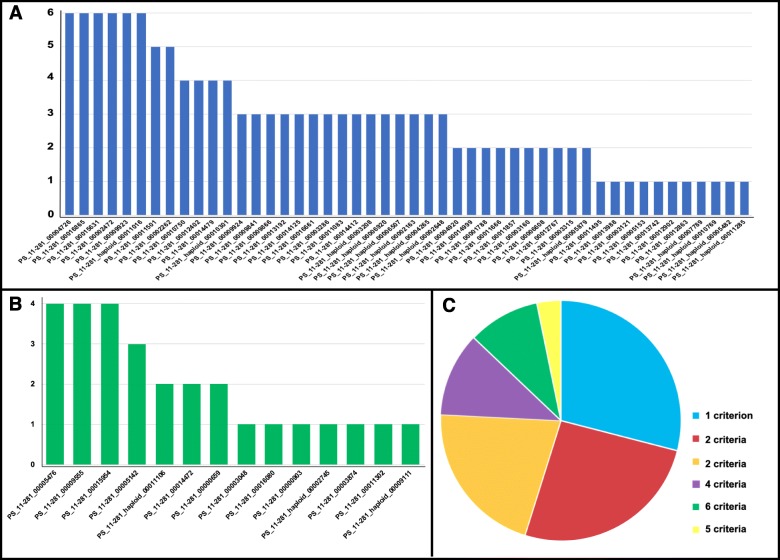
Statistics of effector candidates based on the effector criteria. In A and B: X axes show the names of effector candidates and Y axes show the numbers of effector criteria. **a**: Numbers of criteria met by effector candidates derived from secreted protein (SP) genes. **b**: Numbers of criteria met by effector candidates from non-SP genes with high association (*P* ≤ 0.001). **c**: Pie chart showing the percentages of effector candidates meeting different numbers of criteria

A series of six criteria, including short amino acid sequence, cysteine rich, predicted by EffectorP, genus or species specific, no known domain, and polymorphic within species, were used to evaluate the 62 avirulence gene candidates to obtain effectors with high confidence (Fig. 6). Of the 62 candidates, 11 were predicted to encode small SPs with amino acid length less than 300. Fifteen putative effectors were identified as cysteine-rich proteins with the percentage of cysteine not less than 3%. The avirulence gene candidates were further analyzed using EffectorP, a machine learning fungal effector predictor, and seven of them passed through the criterion and were predicted to be effectors with the possibility greater than 55%. Domains of protein functions were determined by searching the Pfam protein families and InterPro database. No known PFAM domains were found for 37 candidates. Similar results were obtained through searching the InterPro database. Genus and species specific proteins were identified from the orthologous groups, and 34 of the candidates were identified to be *Puccinia* or *P. striiformis* specific proteins through genomic comparison of protein sequences from 13 fungal isolates belonging to 10 species. A phylogenetic tree was generated with these genes using a new rapid hill-climbing algorithm with the GTRGAMMA model. Isolates belonging to ascomycetes and basidiomycetes were assigned to two various clans (Additional file 2: Fig. S4). Isolates of *P. striiformis* were in a cluster closely related to *P. triticina*, *P. graminis* and *P. coronate*; and the wild-type isolate *Pst* 11–281 was tightly clustered with other three *P. striiformis* isolates (*Pst* 104E137A-, *Pst* 93–210 and *Psh* 93TX-2), which have high-quality genomes. Of the 34 genus or species specific genes, 22 were *Puccinia* specific and 12 *P. striiformis* specific; and four of them were basidiomycete orthologs (Additional file 3: Table SE2). The polymorphisms of candidate effectors were identified by searching the existing *P. striiformis* protein database using Blastp. No effector candidates were found to be a *P*. *striiformis* specific and all the 62 candidate genes were found to be polymorphic to at least one isolate among the four *P. striiformis* isolates with high-quality proteomes (Additional file 3: Table SE3). The numbers of criteria of the 62 candidate genes, which were separated into two groups of either SP genes or non-SP genes but with high association (*P* < 0.001), are shown in Fig. 7a and b, respectively; and summarized in Fig. 7c. Of the 48 SP genes, 6 genes met all six criteria and 2 met five criteria (Fig. 7a, Table 5, Additional file 2: Fig. S5A). Among the 14 non-SP genes with high association (*P* value ≤0.001) to avirulence/virulence phenotypes, 3 met four and 1 met three criteria, and the rest 10 met one or two criteria (Fig. 7b, Table 6, Additional file 2: Fig. S5B). These candidates derived from high-degree associated non-SP are more likely to be the irregular or non-effector genes with distinctive characteristics compared with identified effectors.

**Fig. 8:**
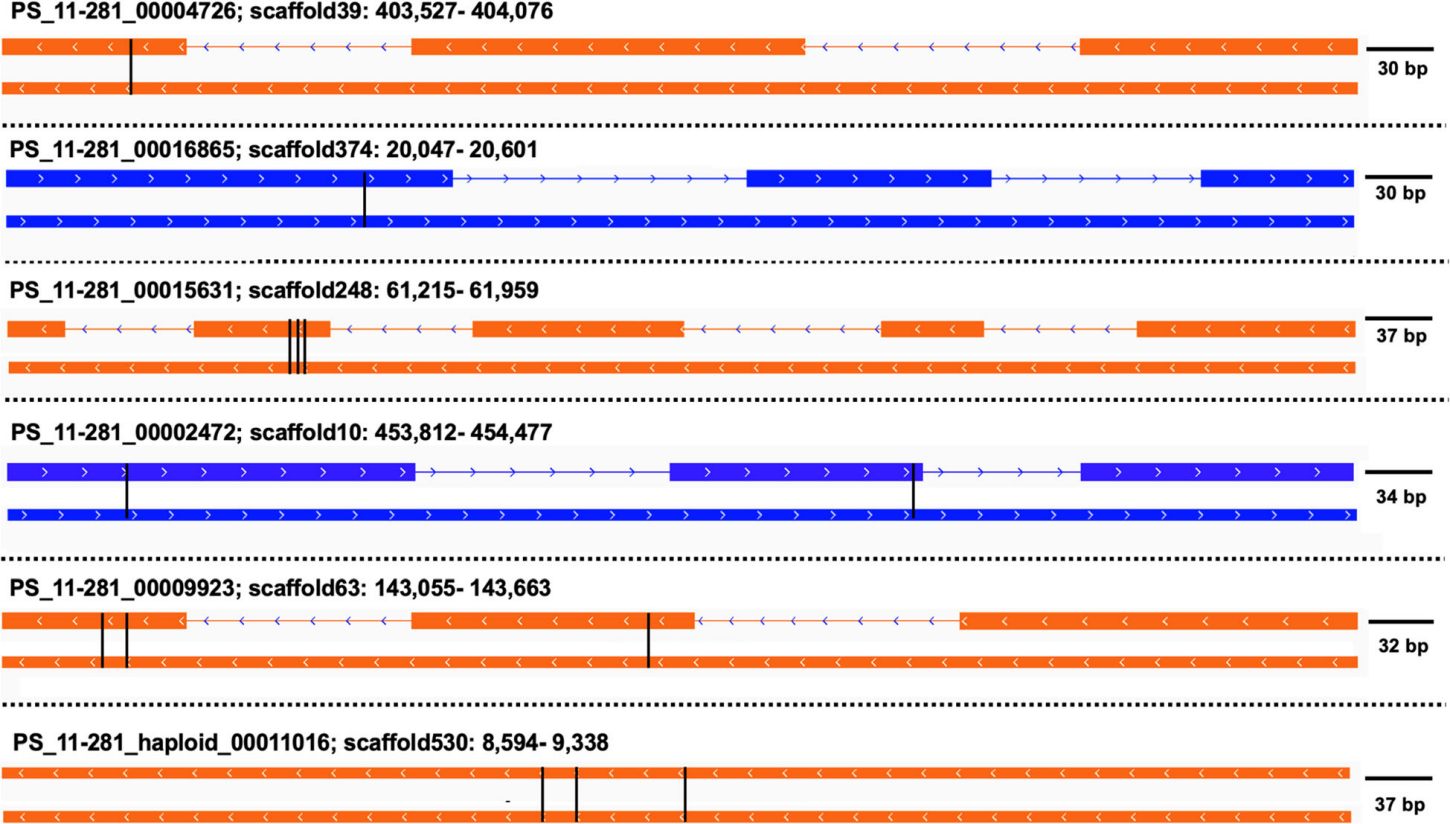
Distribution of deleterious SNPs and Indels in six high-confidence effector genes met all the effector criteria. For each candidate gene, the upper sequence is the gene and the lower sequence shows the exons of the gene. The black lines represent the positions of the SNPs in the gene. For PS_11–281_00004726, the SNPs are in scaffold39: 403,585 (G > A), resulting in Ala > Val. For PS_11–281_00016865, the SNP is in scaffold374: 20,206 (G > A), resulting Val > Ile. For PS_11–281_00015631, the SNPs are in scaffold248: 61,377 (C > T), 61,383 (C > T), and 61,389 (G > A), resulting in Ser > Asn, Gly > Asp, and Ala > Val. For PS_11–281_00002472, the SNPs are in scaffold10: 453,870 (C > T) and 454,477 (C > T), resulting Ala > Val and Thr > Ile. For PS_11–281_00009923, the SNPs are in scaffold63: 143,096 (C > T) and 143,110 (G > A), resulting in Glu > Lys and Ser > Leu. The insertion is in scaffold63: 143,340 (T > TGCTG), resulting in frameshift of Asn. For PS_11–281_haploid_00011016, the SNPs are in scaffold530: 8, 893 (G > A), 8911 (G > A) and 8962 (C > T), resulting His> Tyr, Pro > Ser and Asp >Asn

When the two groups were put together, eight genes met at least five of the criteria and therefore, were considered as candidates for avirulence genes with high confidence. Six of them met all six criteria. Thus, the six genes, PS_11–281_00004726, PS_11–281_00016865, PS_11–281_00015631, PS_11–281_00002472, PS_11–281_00009923 and PS_11–281_haploid_00011016, were predicted to be *Pst* effectors with the highest confidence. Effector gene PS_11–281_00004726 was associated to avirulence loci *AvYr1*, PS_11–281_00016865 was associated to *AvYr6*, PS_11–281_00015631 was associated to *AvYr7*, PS_11–281_00002472 was associated to *AvYr7*, PS_11–281_00009923 was associated to *AvYr76* and PS_11–281_haploid_00011016 was associated to *AvYr17* (Table 5). The SNP and Indel sites occurred in these six effector genes and the resulting amino acids changes are shown in Fig. 8. Although not fitting all six criteria, two genes (PS_11–281_00011501 and PS_11–281_00002262) were still considered as *Pst* effectors associated to avirulence with high confidence as they met five of the six standards. Despite meeting fewer than five effector standards, the rest of 54 candidates were still possible avirulence candidates, and worthy to be included in functional studies.

Four effector motifs, RXLR, [R/K/H] x [L/M/I/F/Y/W]x, [L/I] xAR and [Y/F/W]xC, were found in nineteen putative effectors. Except for PS_11–281_00015631, all these effector candidates contained [Y/F/W]xC and/or RXLR-Like motifs. All the motifs were found within 100 bp from N terminal of each candidate (Table 5, Table 6). Subcellular localizations of the putative effectors in *Pst* were predicted using software WoLF_PSORT. The putative SPs effectors were predicted to be localized mainly in the extracellular spaces of the pathogen (Additional file 2: Fig. S6A), whereas the putative non-SP proteins highly associated with avirulence were predicted to be mostly situated in the nuclei of the pathogen (Additional file 2: Fig. S6B). When the two groups were put together, the majority of gene products were located in the extracellular (47%) and nuclear (29%) spaces of the pathogen (Additional file 2: Fig. S6C). The subcellular localizations effectors inside host plant cells during infection were also predicted. Different from the SP effector candidates (Additional file 2: Fig. S7A), the non-SP candidate products were more likely to target at mitochondria (Additional file 2: Fig. S7B). When the two groups were put together, apoplasts (40%) and nuclei (31%) in host cells are the major targets for the candidate gene products during the process of infection, followed by chloroplasts (16%) and mitochondria (13%) (Additional file 2: Fig. S7C).

## Discussion

It is well-known that mutation is the ultimate source causing genetic variation, resulting in the generation of new alleles and genotypes [50]. The novelty of the present study is that we developed the mutants from EMS mutagenesis and identified the mutation sites throughout the genome, which led to identification of *Avr* effector gene candidates. We expanded the research to the genomic analyses from the previous studies on mutant development and characterization of mutant isolates using virulence testing and molecular markers [10], as well as sequencing the progenitor isolate [49]. By genome sequencing of 30 mutant isolates and comparing with the wild-type isolate genome as the reference to find mutated genes, association analyses and effector characterization, we identified 62 *Pst* effectors with certain levels of high-confidence.

The high-quality assembly and annotation of progenitor isolate genome is the foundation for variation calling. To ensure the premium level of the reference genome, the wild-type isolate was sequenced using both Illumina and PacBio sequencing platforms [49]. The annotation was fulfilled with the help of transcript data retrieved from RNA-seq from different time points. The assessment of completeness showed the high-level of assembly and annotation. In the present study, variant callings were implemented by aligning mutant sequences to the reference genome. We only selected C/G-to-T/A mutations from the variants since a plenitude of EMS mutagenesis studies demonstrated that EMS largely makes C to T and G to A transitions. Previous studies reported a frequency of 92% G/C-to-A/T transitions observed in *Caenorhabditis elegans* [51], 100% in *Drosophila melanogaster* [52] and 99% in Arabidopsis [11]. EMS mutagenesis on other non-model organisms, such as legume [15], rice and wheat [16] and tomato [53], also indicated that EMS induced a biased spectrum of G/C-to-A/T transitions. Thus, the other types of mutations were filtered out in this study, which is the same strategy used on the mutation screening work on Arabidopsis [54], tomato [55], and fungal pathogen *Pgt* [20].

In the genomic studies of *Pst*, most assembled genomes generated a single set of contigs regardless of dikaryotic spore stages. Until recent years, four published genomes of *P*. *striiformis* were assembled into primary contigs and haplotigs [36, 38, 48]. Haplotigs were assembled from divergent regions, which contained SNPs and structural variants, in comparison with the primary contigs [56]. Assembled haplotigs tend to be more fragmented and cannot be simply considered as the genome for the other nucleus. However, omission of haplotigs in the genome analysis may miss some important information. Due to the lack of phased assembles as reference genomes, previous association studies to identify effector candidates on *Pst* and *Pt* focused only on primary contigs [39, 40]. In the present study, we detected SNPs and Indels from both primary scaffolds and haplotigs and conducted association analysis using all identified variants.

In the present study, 67,913 SNPs and 12,886 Indels were detected from the primary scaffolds while 24,796 SNPs and 15,468 Indels from the haplotigs. Thus, the frequency of mutation was 6.40 × 10^− 4^ in term of SNPs and 1.96 × 10^− 4^ in term of Indels. These values are slightly higher but comparable to previously reported 10^− 4^ to 10^− 5^ frequencies of EMS mutation [16, 51, 57]. As the *Pst* mutant isolates were obtained through selection based on moderate to severe changes in virulence, such strong phenotypic selection increased the possibility of detecting variants throughout the pathogen genome. Additionally, the differences in mutation rate may due to the differences in organisms and duration or doses of EMS treatment. The high proportion (70.92 to 99.07%) of mutation SNPs was heterozygous (Table 1). This can be explained by the property of *Pst* and EMS mutagenesis. i) The two nuclei in *Pst* urediniospores were found to be highly heterozygous in many isolates [37, 39, 58, 59]. ii) Previous studies observed *Pst* isolates with a low virulence spectrum tended to be highly heterozygous [39, 60]. The narrow virulence spectrum of *Pst* 11–281 is likely related to its high heterozygosity. iii) The possibility of mutagenesis occurs on the two nuclei to generate homozygous SNPs are much lower than that of producing heterozygous SNPs. Therefore, it is not surprising that heterozygous SNPs took a high proportion of the total EMS-induced SNPs. The number of SNPs and Indels varied greatly among the *Pst* scaffolds. For example, more SNPs were found in primary scaffolds 143, 180, 133 and 277 with the SNP density > 6000 SNP/Mb, whereas no SNPs were found in primary scaffolds 48, 149 and 343. Similarly, the most intensive Indels were found in primary scaffold 370, but no Indels were detected in primary scaffolds 48, 149 and 343 (Fig. 2, Additional file 1: Table S3). The variants densities indicated that mutations occurred from the selected 30 mutant isolates were not randomly distributed, which allowed us to identify genes with SNPs and/or Indels associated to all possible avirulence/virulence phenotypes.

We conducted association analysis between EMS-induced variants and the avirulence/virulence phenotypes based on the IT data of the 30 mutant isolates on each of the 18 *Yr* single-gene lines used to differentiate *Pst* races [10, 48, 61]. SNPs annotated with high and moderate effects were believed to be deleterious, which consisted of splice, missense, start loss and stop gain variants. Likewise, Indels annotated with high and moderate effects were regarded as deleterious Indels, including frameshift, splice, conserved and disruptive inframe Indels, stop codon gained and lost, start codon and exon loss and bidirectional gene fusions. We noticed that deleterious effects counted for 4.2 and 4.6% of the total effects from SNPs and Indels, respectively. Fourteen genes involved in deleterious SNPs and Indels with high association (*P* ≤ 0.001) regardless SPs were identified as effectors or not from a different perspective. Taking association analysis and EMS-induced variant annotation into consideration, a total of 62 genes, including 48 SPs and 14 non-SPs, obtained from the present study were considered to be *Avr* candidates with various degrees of confidence.

There is no a one-size-fits-all standard to predict effectors, but the features of most known fungal effectors are useful for assessing effectors. Effectors have been studied in both biotrophic and necrotrophic fungal pathogens [62–65]. The pipelines to determine rust effector candidates were accomplished by Saunders et al. [41] and Sperschneider et al. [42, 43]. These pipelines are developed based on the observed properties of known effectors in rust fungi, including secreted, small size, cysteine rich and rust-specific. In the present study, we firstly evaluated the 48 *Avr* candidates of SPs based on the six criteria and found them met at least one of the criteria. Eight of the candidates were satisfied with at least five criteria and therefore, considered as high-confidence effector candidates. It is noted that not all the identified avirulence genes encoding SPs. For example, *Magnaporthe oryzae* (*M. oryzae*) *Avr* gene *ACE1* is predicted to be non-secreted and localizes in the cytoplasm of appressoria [66]. Therefore, the 14 non-SP candidates we obtained from variants with high associations to avirulence were evaluated following the same effector standards.

Previous studies showed several cloned *Avr* effectors by meeting the requirements in effector prediction. For example, the *Pgt* effector gene *AvrSr35* encodes a 578–amino acid protein [20], which is greater than the 300 amino acids threshold. The *Bgh* effector *BEC1019* was escaped from the detection with the EffectorP program [67]. Thus, the candidates met more criteria have a higher priority in the future functional verification, but the candidates received relatively low confidence values should not been discarded.

For all the identified effector candidates, sixteen of them were derived from haplotigs of the wild-type isolate, including PS_11–281_haploid_00011016 with a high-degree confidence. Therefore, studies on haplotigs made a significant contribution to identification of effectors in *Pst*. Among all 62 candidates identified, 46 each are associated with one *Avr* locus and 16 each associated to multiple *Avr* loci. For example, the six candidates (PS_11–281_00004726, PS_11–281_00016865, PS_11–281_00015631, PS_11–281_00002472, PS_11–281_00009923 and PS_11–281_haploid_00011016) that met all six criteria each were associated with a single *Avr* locus (*Avr1*, *Avr6*, *Avr7*, *Avr7*, *Avr76* and *Avr17*, respectively). This kind of *Avr* candidates corresponding to only one *R* gene fits the gene-for-gene hypothesis [22]. Therefore, those genes can be considered as *Avr1*, *Avr6*, *Avr7*, *Avr76* and *Avr17* candidates. In a different case, PS_11–281_00011501, which met five of the six criteria, was associated with both *AvYr32* and *AvYrSP*. Interestingly, the avirulent/virulent phenotypes of these two genes are highly correlated among the *Pst* races (6, 60, 61). The shared candidate gene and correlated phenotypes indicate that *AvYr32* and *AvYrSP* are located in a small genomic region, or they are the same avirulence gene, but corresponding to both wheat resistance genes *Yr32* and *YrSP*. It is not unusual in rust fungal effectors. In the cloned effectors of *M. lini*, *AvrL567* corresponds to flax resistance genes *L5*, *L6* and *L7* [28]; and the Avr protein of *AvrM14* is recognized by both *M1* and *M4* resistance proteins in flax [18]. The similar phenomenon was also found in the *Leptosphaeria maculans* effector gene *AvrLm4–7*, which triggers resistance responses to *Rlm4* and *Rlm7* [68]. The observations of one effector gene correlated to multiple *Avr* genes could also be explained by linkages of *Avr* loci. Using a sexually reproduced *Pst* segregating population, Yuan et al. [9] mapped several *Avr* genes to a single chromosomal region.

Effector candidate PS_11–281_00011501 did not meet all the six criteria, but its 307-amino acid length was very close to the threshold of 300 amino acids. Effectors with amino acids greater than 300 were also observed in *Pgt* effector AvrSr35 [20] and *M. lini* effector AvrM [69]. PS_11–281_00002262 fit five criteria but did not pass through program EffectorP 2.0. Similar result was reported for *Bgh* effector CSEP0105, which was also a small size and cysteine-rich protein but failed to be identified as an effector by the EffectorP program [67].

Previous studies failed to identify conserved sequence motifs like oomycetes motif RxLR in fungal effectors [44]. However, few studies found some motifs within a species [46, 47]. In the present study, we identified four motifs in nineteen effector candidates (Table 5, Table 6). It is worth mentioning that motif [Y/F/W]xC was found within 100 bp from the cleavage sites of seven putative effectors with high-degree confidence. Motif [Y/F/W]xC has been detected in SPs in *Pgt* [46, 47] and predicted *Pst* candidates [39]*.* Due to the limited number of cloned effectors in the *Puccinia* species, we could not determine whether [Y/F/W]xC is a conserved motif of effectors in the genus *Puccinia*. RXLR and RXLR-like motifs, which have been commonly found in oomycetes effector, were detected in eight *Pst* avirulence gene candidates in the present study. Also, motif [L/I]xAR previously reported from *M. oryzae* effectors was detected in two of the *Pst* avirulence gene candidates. It is still not clear whether these effector motifs from oomycetes and ascomycetes are related to avirulence genes in *Puccinia* of basidiomycetes. This information is important in characterizing effectors and understanding evolutionary mechanisms of plant pathogenic fungi and oomycetes. Functional verification of more effector candidates in *Puccinia* species and other fungi or oomycetes, conserved motifs could be identified in the near future.

Effectors can be classified into apoplastic, cytoplasmic and nuclear categories based on their subcellular localization and action inside hosts [70]. Since software LOCALIZER can only predict the location of chloroplast, mitochondria and nuclei [71], those with their targets unpredictable were considered as apoplastic effectors. In the present study, 25 avirulence gene candidates were predicted to be apoplastic effectors, with putative functions of inhibiting host proteases and peroxidases. Nineteen effectors were classified as nuclear effectors, which were predicted to suppress the host defense system from the upstream by disturbing gene transcriptions related to host defense. Thirteen effectors were regarded cytoplasmic, which may influence host defense signaling and metabolism by targeting related proteins. Five effectors (PS_11–281_00001788, PS_11–281_00013988, PS_11–281_00013742, PS_11–281_00006608 and PS_11–281_haploid_00003208) were presumed to possess dual functions in both cytoplasm and nucleus, as reported in other fungi [70].

Our study identified 62 *Pst* Avr candidates through comparative genomics and association analyses. With the identification of more effectors in *Puccinia* species in recently years [20, 25, 31, 72], functional verification using RNA silencing and transient expression systems should be plausible approaches to identify *Pst* effectors*.* Confirmation of the roles of these effector candidates in the future studies is very likely to clone *Avr* genes in *Pst*, which should shed light on the mechanisms of interactions between the host and pathogen. Cloning *Avr* genes in the future are important for developing a new approach for quickly detecting *Pst* races in the field and for manipulating the evolution of effectors so as to develop resistance genes and control the disease in a more efficient way.

## Conclusions

In the present study, we further studied 30 mutant isolates developed by EMS mutagenesis of isolate 11–281 of the least virulent race of *Pst* identified so far in the U.S. By genomic sequencing the wild-type isolate and the derived mutants, we aligned the sequences of the mutants with the wild-type isolate to determine sequence variants and identify *Avr* gene candidates. The effects of EMS mutagenesis on *Pst* from the perspective of whole genome were summarized in this study. More importantly, by integrating the virulence data with EMS-induced polymorphisms, we identified 62 candidates for *Avr* genes. In addition, we evaluated and characterized these effector candidates through various bioinformatics analyses and identified eight high-confidence candidates, six of which were meet all the effector criteria. The candidate genes will be used for the upcoming studies to verify their functions. Our study proved that mutagenesis in combination with whole genomic sequencing is a potent approach in identifying high-confidence *Avr* candidates in the obligate biotrophic fungus, leading to unravelling pathogen variation and cloning *Avr* effectors from the stripe rust pathogen in the future.

## Methods

### Selection of mutant isolates

Thirty mutant isolates were selected from those developed through EMS-mutagenesis of *Pst* isolate 11–281, representing PSTv-18, the least virulent race of *Pst* identified so far in the U.S. [48]. These isolates were developed and have been maintained by our stripe rust program in the Wheat Health, Genetics and Quality Research Unit of the US Department of Agriculture, Agricultural Research Service and the Department of Plant Pathology, Washington State University, Pullman, WA, the United States. The isolates were previously tested on the 18 *Yr* single-gene lines used to differentiate *Pst* races [10]. The 30 isolates were selected based on their avirulence/virulence patterns on 16 of the 18 *Yr* single gene lines on which they produced both avirulent and virulent reactions in relatively balanced ratios among the isolates.

### Sequencing of the reference genome and mutant isolates

The whole genome of the progenitor isolate (11–281) sequenced, assembled and annotated as previously reported [49], was used as a reference genome in this study. The assembled genome sequence of *P. striiformis* isolate 11–281 is available on GenBank under the accession number SBIN00000000. The version described in this article is version SBIN01000000. The raw data of isolate 11–281 used for this experiment has been submitted to NCBI with SRA study SRR8446446. Urediniospores of the 30 mutant isolates were collected from infected wheat plants grown in growth chambers, dried in a desiccator at 4 °C for about a week, and used for extracting genomic DNA using the cetyl trimethylammonium bromide (CTAB) method with modifications as previously described [73]. The genomic DNA samples of the 30 mutant isolates were sequenced using the Illumina Hiseq 2000 technology by Novogene Corporation (Sacramento, CA, U.S.A.). The raw data of the 30 mutant isolates have been deposited in the NCBI under BioProject accession PRJNA587768 (containing SRA accessions SRR10413520 to SRR10413549) with the title “Puccinia striiformis f. sp. tritici strain:PST-mutants | isolate:PST-mutants_M11 Raw sequence reads”.

### Sequence alignment and variant detection

After examining the quality of raw paired-ends reads from Illumina sequencing using software FastQC version 0.11.6, the low-quality reads were filtered out using Trimmomatic version 0.36 [74]. The progenitor reference genome was indexed using Burrows-Wheeler Alignment (BWA) Tool version 0.7.17**.** Quality-checked sequences from the 30 mutant isolates were aligned to the reference genome using the BWA-mem algorithm with default options [75]. The SAM formatted file was obtained from alignment and converted to a BAM formatted file using the SAMtools version 1.9 view command. After cleaning unmapped reads, sorting, validating and removing duplicates with Picard tools version 2.18.11, the BAM file was indexed using SAMtools as the input for the Genome Analysis Toolkit (GATK) version 4.1.0.0 HaplotypeCaller to call variants. The GTAK HaplotypeCaller was used twice to call variants in the pipeline. In the first round of calling variants, only SNPs and Indels with a high degree of confidence were kept using VCFtools version 0.1.17 with the threshold value as following: --min-alleles 2 --max-alleles 2 --minQ 1000. The high-confidence SNPs and Indels were used as known variant sites to build a model of covariation, and then they were adjusted for the base quality scores on the basis of the model using BaseRecalibrator. The base quality scores were recalibrated using ApplyBQSR to generate recalibrated BAM files that served as input files for the second round variant calling using GTAK HaplotypeCaller. The SNPs and Indels were selected based on the parameters setting as: --min-alleles 2 --max-alleles 2 --minQ 30 --min-meanDP 5 --max-meanDP 90 --max-missing 1. In addition, hard-filtering was performed to filter the variants and select the high-quality SNPs and Indels using VariantFiltration. The thresholds setting as: “QUAL < 0 || MQ < 40.00 || SOR > 4.000 || QD < 2.00 || FS > 60.000 || MQRankSum < -20.000 || ReadPosRankSum < -10.000 || ReadPosRankSum > 10.000” in SNPs filtering and “QUAL < 0 || MQ < 40.00 || SOR > 10.000 || QD < 2.00 || FS > 200.000 || ReadPosRankSum < -20.000 || ReadPosRankSum > 20.000 || DP >= 20” in Indel filtering, which were recommended in the GATK “best practice” with modifications (https://software.broadinstitute.org/gatk/best-practices/). SNPs were further filtered by selecting only C to T and G to A mutations since mutations caused by EMS had the strong CG to TA transition bias [11, 15, 16, 47, 53].

In order to eliminate SNPs and Indels from heterozygosity of *Pst* 11–281, we used the Illumina sequenced data of 11–281 to control SNP and Indel callings. Alignment of the Illumina data to the assembled reference genome followed above mentioned BWA-mem and GATK procedures. The variants from all 30 mutants were filtered by removing heterozygous sites of SNPs and Indels from both the primary scaffolds and haplotigs.

### Characterization of genomic variation in mutants

The effect of SNPs and Indels concerning gene functions were determined separately using the program SnpEff version 4.3 t [76]. To begin with SnpEff, the *Pst* 11–281 genome database was built via inputting the assembled genome and GFF3 formatted file derived from MAKER2. Variants were annotated using the database we built under the default settings. The mutations were assigned to impact categories from the SnpEff analysis, including high, moderate, modifier and low variants. The associated amino acid variations and whether they are in accordance with deleterious mutations were also detected using SnpEff. Only the deleterious mutations with high and moderate impacts, e.g. start lost, stop gained and missense variant, frameshift variants, etc., were selected for the further identification. Mutations with modifiers and low effect impacts, which did not impact directly on the genetic variants, were excluded.

### Association analysis between mutations and avirulence/virulence phenotypes

Genes from isolate 11–281 with deleterious mutations were extracted from the annotated VCF formatted file from the SnpEff outputs. Association between mutated genes and avirulence/virulence phenotypes were analyzed using genome association and prediction integrated tool (GAPIT version 3.0) in the R program [77, 78]. The recorded ITs in numeric and mutated genes converted to numeric formats were used as phenotypic and genotypic inputs for the GAPIT analysis. The mixed linear model (MLM), which incorporated fixed and random effects, was implemented in the association analysis. SNPs and Indels were considered significantly or highly associated with avirulence/virulence phenotypes if *P* value ≤0.05 or *P* value ≤0.001, respectively.

### Identification of *Avr* candidates

The associated genes and their predicted proteins were obtained from the GAPIT analysis. SPs were predicted from the proteins following the previously described pipeline for determining rust secretomes [43]. Proteins with signal peptides, which are presumed to have extracellular functions, were identified using SignalP 5.0 [79]. Proteins with predicted sequences containing transmembrane helices were excluded from the set of predicted extracellular proteins using TMHMM version 2.0 [80]. Selected SPs associated with avirulence/virulence phenotypes, which also had the amino acid changes, were identified as *Avr* candidates. In addition, genes obtained from high association with *P* value ≤0.001 regardless SPs were predicted to be another set of putative effectors.

### Characterization of *Avr* genes candidates

To characterize *Avr* effector candidates, multiple criteria that have been commonly used to identify effectors were used. These criteria include: 1) the size of amino acid sequence less than 300; 2) cysteine-rich with the percentage equal to or greater than 3%**;** 3) predicted by the program EffectorP; 4) genus or species specific; 5) no conserved domains and 6) polymorphic among *P. striiformis* isolates*.*

The amino acid lengths and cysteine percentages of candidate Avr effectors were calculated using a modified Python script [43]. Fungal effector prediction in secretomes were implemented using EffectorP version 2.0, which utilizes different approaches to predict effectors based on a large set of reported effectors [67]. In order to identify homologous relationships between sequences to generate orthologous groups, thirteen protein sequences in ten species, including *Pst*, *P. striiformis* f. sp. *hordei* (*Psh*, the barley stripe rust pathogen), *P. triticina* (*Pt*, the wheat leaf rust pathogen), *Pgt*, *Puccinia coronata* f. sp. *avenae* (*Pca,* the oat crown rust pathogen), *Blumeria graminis* f. sp. *hordei* (*Bgh*, the barley powdery mildew pathogen), *Botrytis cinerea* (*Bc*), *Fusarium oxysporum* f. sp. *cepae* (*Foc*), *Fusarium graminearum* (*Fg*), *Melampsora larici-populina* (*Mlp*) and *Verticillium dahliae* (*Vd*), were used to estimate the phylogenetic relationships using OrthoFinder version 2.3.1 [81]. A phylogenetic tree was inferred based on the orthogroups of each isolate, and an orthologous group number was assigned to each of the proteins. According to the orthologous groups of the Avr effector candidates, the genus-specific and species-specific proteins were identified. Since known obligated biotrophic effectors rarely have known functional domains [45], absence of domains is considered as one property of effectors. In this study, we assigned functional domains to the effector candidates using HMMSCAN searches against the PFAM protein family database using HmmerWeb version 2.36.0 [82] and searching InterPro database (http://www.ebi.ac.uk/InterProScan/). To identify polymorphic candidate effectors, eighteen candidates were searched for homologs from four existing *Ps* protein database (104E137A-, 93–210, 93TX-2 and PST-78) using BLAST (basic local alignment search tool) version 2.7.1+ with the E-value 1e-50, which stands for the extremely high confidence that the database match is a result of homologous relationships. Only the candidates found in all the four protein databases with 100% identity, no mismatch and no gaps detected were considered as monomorphism among *Pst* and otherwise, candidates were deemed to be polymorphic.

### Searching motifs and subcellular localizations of effectors in *Pst* and in the host

**Fig. 9:**
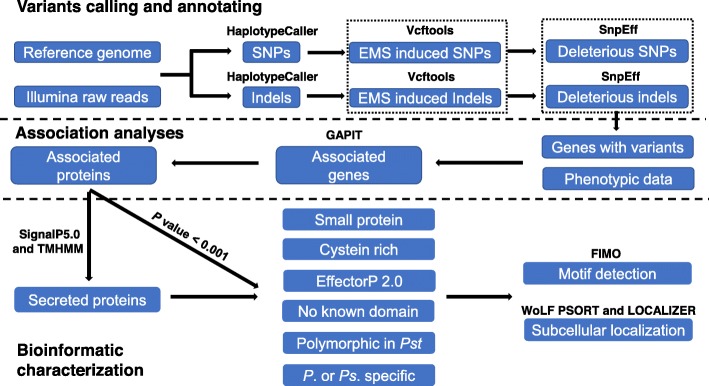
A schematic procedure for variants calling and annotating, association analysis and effector evaluation. Variants calling and annotating were implemented mainly using GATK HaplotypeCaller and SnpEff, respectively. The output files were integrated with phenotypic data to fulfill association analyses using GAPIT. The *Avr* effector candidates obtained from selected associated proteins were further evaluated and characterized in different aspects

Associated secreted proteins were searched for common effector motifs, including RxLR [44], RxLR-like motif [R/K/H]x[L/M/I/F/Y/W]x [83] and YxSL[R/K] [84] detected in oomycetes, [L/I]xAR and [R/K]CxxCx12H [85] in some effectors of the rice blast pathogen (*M. oryzae*), [R/K]VY[L/I]R [86] in *Bgh*, [Y/F/W]xC [46] in the wheat powdery mildew and rust effector candidates and G[I/F/Y][A/L/S/T]R [27] in some effectors of the flax rust pathogen (*M. lini*). Motifs were scanned using FIMO version 5.1.1 in the MEME suite with a match *P* value less than 0.0001 [87]. The subcellular localizations were predicted by amino acid sequence searching of the Fungi databases using the WoLF PSORT program (https://wolfpsort.hgc.jp/). The localizations of effectors in plant cells were predicted using the program LOCALIZER version 1.0.4, which determines effector targets as in chloroplasts, mitochondria or nuclei using the mature effector sequences [43]. The workflow of variant calling, association analysis, effector identification and evaluation were presented in the Fig. 9.

## Supplementary information

**Additional file 1: Table S1.** Infection types of the progenitor isolate 11–281 and derived 30 mutants of *Puccinia striiformis* f. sp. *tritici* on 18 wheat *Yr* single-gene differentials. **Table S2.** Reads, rates and qualities of 30 *Puccinia striiformis* f. sp. *tritici* mutants mapped to the reference genome. **Table S3.** The distribution and density of SNPs and Indels on each scaffold. **Table S4.** Statistics of Indels of each mutant isolate in terms of insertions, deletions, and different lengths. **Table S5.** Number of SNPs, Indels, insertions and deletions of each mutant isolate identified from the isolate 11–281 haplotigs. **Table S6.** Genes highly associated to avirulence with *P*-value < 0.001.

**Additional file 2: Figure S1.** Phylogenetic trees of *Puccinia striiformis* f. sp. *tritici* mutant isolates based on the EMS-induced SNPs. **Figure S2.** Types and frequencies of SNP effects detected from the reference genome haplotigs. A: The number and percentage of all EMS-induced SNPs for each type of effects. B: The types and percentages of deleterious SNP effects. **Figure S3.** Types and frequencies of Indel effects detected from the reference genome haplotigs. A: The number and percentages of all EMS-induced Indels for each type of effects. B: The types and percentages of deleterious Indel effects. **Figure S4.** Phylogenetic tree of the progenitor isolate 11–281 of *Puccinia striiformis* f. sp. *tritici* (*Pst*) together with other 12 fungal isolates. The phylogenetic tree was developed from 13 protein sequences using a hill-climbing algorithm with the GTRGAMMA model. 13 fungi isolates are *Verticillium dahlia* 12,008, *Fusarium oxysporum* f. sp. *cepae* FoC_Fus2, *Fusarium graminearum* ITEM_124, *Blumeria graminis* f. sp. *hordei* RACE1, *Botrytis cinerea* B05.10, *Melampsora larici-populina* 98AG31, *Puccinia striiformis* f. sp. *tritici* 104E137A-, 93–210, 11–281, *Puccinia striiformis* f. sp. *hordei* 93TX-2, *Puccinia coronata* f. sp. *avenae* 12SD80, *Puccinia graminis* f. sp. *tritici* CRL75–36–700-3 and *Puccinia triticina* BBBD Race 1. The numbers indicate the distances between nodes. **Figure S5.** Characterization of *Puccinia striiformis* f. sp. *tritici Avr* effector candidates in the Upset plot. A: 48 effector candidates of secreted protein (SP) genes. B: 14 effector candidates from non-SP genes. **Figure S6.** Percentages (%) of the subcellular localization sites of effector candidates of *Puccinia striiformis* f. sp. *tritici* in the fungal cells. A: Genes identified from secreted protein (SP) genes. B: Genes identified from non-SP genes. C: All 62 genes associated to avirulence genes. **Figure S7.** Percentages (%) of the subcellular localization sites of effector candidates of *Puccinia striiformis* f. sp. *tritici* inside the host. A: Genes identified from secreted protein (SP) genes. B: Genes identified from non-SP genes. C: All 62 genes associated to avirulence genes.

**Additional file 3: Table SE1.** 62 genes considered as candidates for avirulence genes and their genome positions and derived amino acids**. Table SE2.** Orthogroups of *Puccinia striiformis* f. sp. *tritici* (*Pst*) avirulence gene candidates in isolate *Pst* 11–281 detected in other 12 fungal isolates. **Table SE3.** Search results of candidate genes identified in *Puccinia striiformis* f. sp. *tritici* (*Pst*) isolate *Pst* 11–281 against four *P. striiformis* protein databases using NCBI BLAST.

## Data Availability

The whole genome of the progenitor isolate (11–281) sequenced, assembled and annotated as previously reported [49], was used as a reference genome in this study. The assembled genome sequence of *P. striiformis* isolate 11–281 is available on GenBank under the accession number SBIN00000000. The version described in this article is version SBIN01000000. The raw data of isolate 11–281 used for this experiment has been submitted to NCBI with SRA study SRR8446446. The raw data of the 30 mutant isolates have been deposited in the NCBI under BioProject accession PRJNA587768 (containing SRA accessions SRR10413520 to SRR10413549) with the title “Puccinia striiformis f. sp. tritici strain:PST-mutants | isolate:PST-mutants_M11 Raw sequence reads”. All analyzed data are provided in the additional files of this publication.

## Abbreviations

*Avr*: Avirulence gene
BLAST: Basic local alignment search tool;
BWA: Burrows-wheeler alignment
CTAB: Cetyl trimethylammonium bromide
EMS: Ethyl methanesulfonate
ETI: Effector-triggered immunity
GATK: Genome analysis toolkit
Indels: Insertion/deletion
IT: Infection type
MLM: Mixed linear model
NCBI: National center of biological information
*P*: Probability
PAMP: Pathogen-associated molecular pattern
*Pgt*: *Puccinia graminis* f. sp. *tritici*
*Ps*: *Puccinia striiformis*
*Psh*: *Puccinia striiformis* f. sp. *hordei*
*Pst*: *Puccinia striiformis* f. sp. *tritici*
*Pt*: *Puccinia triticina*
PTI: PAMP triggered immunity
R: Resistance
SNP: Single nucleotide polymorphism
SP: Secreted protein
*Yr*: Yellow (stripe rust) resistance
