# Supplementary material for: Whole-genome sequencing of Puccinia striiformis f. sp. tritici mutant isolates identifies avirulence gene candidates
Source: BMC Genomics. 2020 Mar 20;21:247. doi: 10.1186/s12864-020-6677-y (PMC7085141; doi:10.1186/s12864-020-6677-y)
Supplement: Supplementary file 1 — Additional file 1: Table S1. Infection types of the progenitor isolate 11–281 and derived 30 mutants of Puccinia striiformis f. sp. tritici on 18 wheat Yr single-gene differentials. Table S2. Reads, rates and qualities of 30 Puccinia striiformis f. sp. tritici mutants mapped to the reference genome. Table S3. The distribution and density of SNPs and Indels on each scaffold. Table S4. Statistics of Indels of each mutant isolate in terms of insertions, deletions, and different lengths. Table S5. Number of SNPs, Indels, insertions and deletions of each mutant isolate identified from the isolate 11–281 haplotigs. Table S6. Genes highly associated to avirulence with P-value < 0.001. [file 12864_2020_6677_MOESM1_ESM.docx]

**Additional File 1 (Tables S1-S6)**

**Table S1** Infection types of the progenitor isolate 11-281 and derived 30 mutants of *Puccinia striiformis* f. sp. *tritici* on 18 wheat *Yr* single-gene differentials

|  | Infection types on 18 *Yr* single-gene differential lines^a^ | | | | | | | | | | | | | | | | | |
| --- | --- | --- | --- | --- | --- | --- | --- | --- | --- | --- | --- | --- | --- | --- | --- | --- | --- | --- |
| Isolate | *Yr1* | *Yr5* | *Yr6* | *Yr7* | *Yr8* | *Yr9* | *Yr10* | *Yr15* | *Yr17* | *Yr24* | *Yr27* | *Yr32* | *Yr43* | *Yr44* | *YrSP* | *YrTr1* | *YrExp2* | *Yr76* |
| 11-281  (Wild type) | 2 | 1 | 2 | 2 | 2 | 2 | 1 | 1 | 1 | 1 | 2 | 2 | 1 | 2 | 1 | 2 | 1 | 2 |
| M11-Yr1-1 | 7 | 1 | 2 | 1 | 2 | 8 | 1 | 1 | 6 | 2 | 2 | 2 | 2 | 2 | 8 | 1 | 2 | 8 |
| M11-Yr1-2 | 8 | 1 | 1 | 8 | 7 | 8 | 1 | 1 | 6 | 2 | 7 | 5 | 4 | 8 | 8 | 7 | 2 | 8 |
| M11-Yr1-3 | 8 | 1 | 2 | 4 | 5 | 8 | 1 | 1 | 6 | 2 | 7 | 5 | 4 | 8 | 8 | 7 | 2 | 8 |
| M11-Yr2-1 | 8 | 1 | 2 | 2 | 2 | 8 | 2 | 1 | 5 | 2 | 7 | 2 | 2 | 7 | 7 | 2 | 2 | 8 |
| M11-Yr2-2 | 8 | 1 | 2 | 2 | 2 | 8 | 1 | 1 | 7 | 2 | 7 | 2 | 2 | 2 | 8 | 1 | 2 | 8 |
| M11-Yr6 | 1 | 2 | 8 | 8 | 7 | 7 | 7 | 1 | 4 | 8 | 6 | 7 | 7 | 8 | 1 | 8 | 8 | 2 |
| M11-Yr8 | 2 | 1 | 2 | 2 | 2 | 1 | 1 | 1 | 1 | 1 | 1 | 1 | 1 | 2 | 1 | 1 | 2 | 2 |
| M11-Yr9-1 | 8 | 2 | 8 | 8 | 7 | 7 | 2 | 1 | 7 | 2 | 7 | 2 | 7 | 8 | 1 | 2 | 8 | 7 |
| M11-Yr9-2 | 8 | 1 | 2 | 8 | 8 | 8 | 1 | 1 | 8 | 2 | 8 | 2 | 8 | 8 | 2 | 8 | 8 | 3 |
| M11-Yr9-4 | 8 | 1 | 7 | 2 | 2 | 1 | 1 | 1 | 5 | 1 | 7 | 2 | 2 | 2 | 8 | 1 | 2 | 8 |
| M11-Yr10 | 1 | 1 | 8 | 8 | 8 | 8 | 8 | 1 | 7 | 8 | 8 | 8 | 7 | 8 | 1 | 8 | 8 | 1 |
| M11-Yr17 | 8 | 1 | 8 | 2 | 7 | 8 | 1 | 1 | 6 | 2 | 8 | 2 | 6 | 7 | 8 | 7 | 2 | 8 |
| M11-Yr21 | 8 | 1 | 8 | 6 | 5 | 8 | 1 | 1 | 7 | 2 | 8 | 2 | 5 | 7 | 8 | 5 | 7 | 8 |
| M11-Yr24-1 | 1 | 1 | 2 | 8 | 8 | 8 | 1 | 1 | 8 | 2 | 8 | 1 | 8 | 8 | 1 | 7 | 8 | 1 |
| M11-Yr31 | 1 | 1 | 2 | 2 | 2 | 1 | 1 | 1 | 1 | 1 | 1 | 1 | 1 | 1 | 1 | 1 | 1 | 2 |
| M11-Yr36-1 | 7 | 1 | 8 | 7 | 1 | 7 | 7 | 1 | 2 | 2 | 7 | 7 | 2 | 7 | 7 | 7 | 2 | 1 |
| M11-Yr36-2 | 8 | 1 | 1 | 3 | 1 | 8 | 1 | 1 | 2 | 2 | 7 | 2 | 3 | 2 | 8 | 8 | 3 | 8 |
| M11-Yr39 | 1 | 1 | 8 | 8 | 8 | 8 | 8 | 1 | 7 | 8 | 7 | 7 | 8 | 8 | 1 | 8 | 8 | 1 |
| M11-Yr43 | 1 | 1 | 3 | 8 | 8 | 8 | 2 | 1 | 8 | 1 | 7 | 2 | 8 | 7 | 1 | 2 | 1 | 2 |
| M11-Yr44 | 2 | 2 | 2 | 8 | 2 | 2 | 7 | 1 | 1 | 8 | 7 | 2 | 7 | 2 | 1 | 7 | 8 | 2 |
| M11-YrSP-1 | 8 | 1 | 3 | 2 | 2 | 8 | 1 | 1 | 7 | 1 | 7 | 1 | 2 | 2 | 8 | 1 | 3 | 8 |
| M11-YrSP-2 | 8 | 2 | 2 | 2 | 2 | 8 | 1 | 1 | 6 | 2 | 6 | 1 | 3 | 2 | 8 | 1 | 2 | 8 |
| M11-YrTr1 | 1 | 1 | 1 | 1 | 8 | 1 | 1 | 1 | 3 | 1 | 1 | 1 | 1 | 1 | 1 | 1 | 1 | 1 |
| M11-YrExp2 | 8 | 1 | 8 | 2 | 2 | 8 | 1 | 1 | 6 | 2 | 7 | 2 | 2 | 8 | 8 | 1 | 2 | 8 |
| M11-Yr76-1 | 4 | 1 | 2 | 3 | 7 | 1 | 1 | 1 | 1 | 1 | 2 | 2 | 1 | 2 | 1 | 1 | 2 | 3 |
| M11-Yr76-2 | 8 | 1 | 2 | 2 | 2 | 8 | 1 | 1 | 6 | 2 | 7 | 2 | 2 | 8 | 8 | 1 | 2 | 8 |
| M11-Yr76-3 | 7 | 1 | 2 | 3 | 7 | 1 | 1 | 1 | 1 | 1 | 3 | 1 | 1 | 2 | 1 | 1 | 2 | 5 |
| M11-YrA+ | 1 | 1 | 8 | 8 | 7 | 8 | 8 | 1 | 7 | 8 | 7 | 8 | 7 | 8 | 1 | 7 | 8 | 1 |
| M11-Fielder | 1 | 1 | 8 | 8 | 8 | 8 | 8 | 1 | 2 | 8 | 7 | 8 | 7 | 8 | 1 | 8 | 7 | 1 |
| M11-Paha | 8 | 1 | 2 | 2 | 2 | 8 | 1 | 1 | 7 | 1 | 7 | 2 | 2 | 2 | 8 | 1 | 2 | 8 |
| **Freq. (%) of virulence^b^** | **60.0** | **0** | **36.7** | **40.0** | **46.7** | **76.7** | **23.3** | **0** | **36.7** | **20** | **73.3** | **20.0** | **33.3** | **56.7** | **50.0** | **43.3** | **33.3** | **50.0** |

^a^ The wheat Yr single-gene differentials contain *Yr1*, *Yr5*, *Yr6*, *Yr7*, *Yr8*, *Yr9*, *Yr10*, *Yr15*, *Yr17*, *Yr24*, *Yr27*, *Yr32*, *Yr43*, *Yr44*, *YrSP*, *YrTr1*, *YrExp2*, or *Yr76*. Infection types from 0 to 6 were regarded avirulent and 7 to 9 virulent [60].

^b^ The percentage of changes of avirulence to virulence to each *Yr* gene was calculated as the number of isolates with the presence of virulence divided by the total number of mutant isolates (30) times 100.

**Table S2** Reads, rates and qualities of 30 *Puccinia striiformis* f. sp. *tritici* mutants mapped to the reference genome

| Mutant | Number of  reads (bp) | Mapped reads (bp)  on primary contigs | Mapping rate (%)  on primary contigs | Mapped reads (bp) on haplotigs | Mapping rate (%) on haplotigs |
| --- | --- | --- | --- | --- | --- |
| M11-Yr76-3 | 27,007,348 | 17,898,645 | 66.27 | 15,812,453 | 57.13 |
| M11-Yr2-2 | 25,401,995 | 17,851,758 | 70.28 | 15,771,860 | 60.80 |
| M11-Yr31 | 20,874,867 | 14,571,163 | 69.80 | 12,901,404 | 60.37 |
| M11-Yr39 | 26,425,540 | 18,590,254 | 70.35 | 16,441,963 | 60.89 |
| M11-Yr8 | 19,107,903 | 13,381,559 | 70.03 | 11,803,120 | 60.38 |
| M11-Yr43 | 23,253,475 | 15,731,967 | 67.65 | 13,901,887 | 58.39 |
| M11-YrExp2 | 18,758,038 | 13,493,063 | 71.93 | 11,971,829 | 62.50 |
| M11-Fielder | 21,195,183 | 14,856,183 | 70.09 | 13,113,322 | 60.49 |
| M11-Yr1-1 | 25,360,441 | 17,219,542 | 67.90 | 15,203,159 | 58.51 |
| M11-Yr9-1 | 20,749,756 | 14,665,624 | 70.68 | 12,928,002 | 60.92 |
| M11-Yr24-1 | 26,508,740 | 17,858,820 | 67.37 | 15,725,517 | 57.95 |
| M11-YrTr1 | 21,571,018 | 14,099,259 | 65.36 | 12,446,079 | 56.36 |
| M11-Paha | 22,631,145 | 16,092,173 | 71.11 | 14,194,389 | 61.38 |
| M11-Yr76-1 | 21,244,862 | 14,164,235 | 66.67 | 12,459,422 | 57.31 |
| M11-YrA+ | 23,805,395 | 16,788,595 | 70.52 | 14,792,192 | 60.72 |
| M11-Yr9-2 | 20,642,227 | 13,536,504 | 65.58 | 11,931,893 | 56.52 |
| M11-Yr44 | 20,246,081 | 14,341,635 | 70.84 | 12,659,709 | 61.13 |
| M11-Yr2-1 | 23,813,670 | 16,776,544 | 70.45 | 14,811,905 | 60.87 |
| M11-Yr36-2 | 23,816,465 | 16,457,551 | 69.10 | 14,522,107 | 59.64 |
| M11-Yr9-4 | 22,903,688 | 16,260,308 | 70.99 | 14,370,872 | 61.35 |
| M11-Yr1-3 | 19,128,776 | 13,722,197 | 71.74 | 12,124,043 | 62.17 |
| M11-Yr10 | 23,101,628 | 15,774,905 | 68.28 | 13,906,448 | 58.91 |
| M11-Yr76-2 | 20,217,979 | 14,178,297 | 70.13 | 12,531,845 | 60.73 |
| M11-Yr21 | 21,886,339 | 14,533,731 | 66.41 | 12,852,579 | 57.58 |
| M11-Yr36-1 | 19,148,621 | 12,691,227 | 66.28 | 11,224,509 | 57.44 |
| M11-Yr17 | 25,805,893 | 17,834,581 | 69.11 | 15,749,176 | 59.81 |
| M11-YrSP-1 | 19,858,443 | 13,630,815 | 68.64 | 12,066,723 | 59.58 |
| M11-YrSP-2 | 18,544,864 | 12,832,376 | 69.20 | 11,325,106 | 59.84 |
| M11-Yr1-2 | 26,977,145 | 18,284,787 | 67.78 | 16,133,787 | 58.54 |
| M11-Yr6 | 19,873,515 | 13,833,189 | 69.61 | 12,197,169 | 60.16 |
| **Average** | **22,328,701** | **15,398,383** | **69.01** | **15,398,383** | **59.61** |

**Table S3** The distribution and density of SNPs and indels on each scaffold

| Scaffold | Size of scaffold (bp) | SNP Count | SNP/Mb | Indel Count | Indel/Mb |
| --- | --- | --- | --- | --- | --- |
| 1 | 1,453,101 | 4439 | 3055 | 1710 | 1177 |
| 2 | 1,409,985 | 5979 | 4240 | 2450 | 1738 |
| 3 | 1,199,131 | 3833 | 3196 | 1395 | 1163 |
| 4 | 1,188,996 | 4236 | 3563 | 1378 | 1159 |
| 5 | 1,095,618 | 3867 | 3530 | 1187 | 1083 |
| 6 | 1,073,982 | 3075 | 2863 | 979 | 912 |
| 7 | 1,056,591 | 4516 | 4274 | 1440 | 1363 |
| 8 | 987,840 | 3413 | 3455 | 957 | 969 |
| 9 | 964,762 | 2645 | 2742 | 927 | 961 |
| 10 | 939,516 | 3244 | 3453 | 1031 | 1097 |
| 11 | 789,896 | 2513 | 3181 | 889 | 1125 |
| 12 | 778,136 | 3022 | 3884 | 947 | 1217 |
| 13 | 768,061 | 2497 | 3251 | 891 | 1160 |
| 14 | 747,084 | 2159 | 2890 | 676 | 905 |
| 15 | 729,850 | 2954 | 4047 | 1041 | 1426 |
| 16 | 726,554 | 2121 | 2919 | 775 | 1067 |
| 17 | 1,219,475 | 4990 | 4092 | 1373 | 1126 |
| 18 | 722,512 | 2202 | 3048 | 909 | 1258 |
| 19 | 720,873 | 2370 | 3288 | 744 | 1032 |
| 20 | 708,975 | 2378 | 3354 | 639 | 901 |
| 21 | 703,360 | 2411 | 3428 | 804 | 1143 |
| 22 | 694,115 | 2267 | 3266 | 634 | 913 |
| 23 | 678,722 | 5 | 7 | 1 | 1 |
| 24 | 670,749 | 2142 | 3193 | 927 | 1382 |
| 25 | 652,709 | 2128 | 3260 | 938 | 1437 |
| 26 | 644,839 | 1870 | 2900 | 817 | 1267 |
| 27 | 638,956 | 1833 | 2869 | 587 | 919 |
| 28 | 622,436 | 2244 | 3605 | 729 | 1171 |
| 29 | 617,796 | 2045 | 3310 | 757 | 1225 |
| 30 | 616,917 | 1564 | 2535 | 641 | 1039 |
| 31 | 608,670 | 2107 | 3462 | 710 | 1166 |
| 32 | 602,139 | 2015 | 3346 | 697 | 1158 |
| 33 | 599,982 | 1884 | 3140 | 472 | 787 |
| 34 | 591,956 | 2029 | 3428 | 688 | 1162 |
| 35 | 589,806 | 1628 | 2760 | 510 | 865 |
| 36 | 589,685 | 1618 | 2744 | 622 | 1055 |
| 37 | 578,945 | 2348 | 4056 | 877 | 1515 |
| 38 | 569,963 | 2619 | 4595 | 639 | 1121 |
| 39 | 562,102 | 2111 | 3756 | 634 | 1128 |
| 40 | 557,058 | 1658 | 2976 | 676 | 1214 |
| 41 | 552,546 | 2619 | 4740 | 914 | 1654 |
| 42 | 548,663 | 1591 | 2900 | 507 | 924 |
| 43 | 544,811 | 1678 | 3080 | 447 | 820 |
| 44 | 542,989 | 1813 | 3339 | 584 | 1076 |
| 45 | 537,992 | 2256 | 4193 | 588 | 1093 |
| 46 | 523,829 | 1419 | 2709 | 569 | 1086 |
| 47 | 487,156 | 1502 | 3083 | 480 | 985 |
| 48 | 485,291 | 0 | 0 | 0 | 0 |
| 49 | 480,909 | 1238 | 2574 | 460 | 957 |
| 50 | 474,580 | 1394 | 2937 | 603 | 1271 |
| 51 | 473,643 | 1598 | 3374 | 461 | 973 |
| 52 | 473,560 | 1369 | 2891 | 525 | 1109 |
| 53 | 454,678 | 2000 | 4399 | 486 | 1069 |
| 54 | 453,824 | 1485 | 3272 | 585 | 1289 |
| 55 | 453,055 | 1288 | 2843 | 373 | 823 |
| 56 | 451,922 | 1503 | 3326 | 596 | 1319 |
| 57 | 448,238 | 1909 | 4259 | 670 | 1495 |
| 58 | 440,942 | 1233 | 2796 | 287 | 651 |
| 59 | 440,740 | 1139 | 2584 | 518 | 1175 |
| 60 | 434,240 | 1478 | 3404 | 547 | 1260 |
| 61 | 431,832 | 1253 | 2902 | 358 | 829 |
| 62 | 431,340 | 1185 | 2747 | 526 | 1219 |
| 63 | 431,121 | 1156 | 2681 | 307 | 712 |
| 64 | 414,035 | 1109 | 2679 | 357 | 862 |
| 65 | 410,808 | 1342 | 3267 | 403 | 981 |
| 66 | 409,565 | 1103 | 2693 | 310 | 757 |
| 67 | 409,348 | 1409 | 3442 | 461 | 1126 |
| 68 | 407,656 | 1680 | 4121 | 477 | 1170 |
| 69 | 406,826 | 1362 | 3348 | 429 | 1055 |
| 70 | 403,343 | 1279 | 3171 | 360 | 893 |
| 71 | 399,639 | 1114 | 2788 | 284 | 711 |
| 72 | 396,936 | 1670 | 4207 | 781 | 1968 |
| 73 | 395,528 | 1377 | 3481 | 442 | 1117 |
| 74 | 392,170 | 1503 | 3833 | 449 | 1145 |
| 75 | 389,819 | 1030 | 2642 | 411 | 1054 |
| 76 | 388,695 | 1381 | 3553 | 407 | 1047 |
| 77 | 385,547 | 1131 | 2933 | 366 | 949 |
| 78 | 384,653 | 1004 | 2610 | 365 | 949 |
| 79 | 375,549 | 1023 | 2724 | 417 | 1110 |
| 80 | 375,105 | 1270 | 3386 | 469 | 1250 |
| 81 | 374,087 | 1226 | 3277 | 358 | 957 |
| 82 | 369,795 | 992 | 2683 | 420 | 1136 |
| 83 | 366,097 | 1272 | 3474 | 358 | 978 |
| 84 | 365,595 | 1478 | 4043 | 423 | 1157 |
| 85 | 356,997 | 1052 | 2947 | 348 | 975 |
| 86 | 342,969 | 1086 | 3166 | 334 | 974 |
| 87 | 342,708 | 1216 | 3548 | 492 | 1436 |
| 88 | 339,674 | 1122 | 3303 | 256 | 754 |
| 89 | 331,375 | 1456 | 4394 | 424 | 1280 |
| 90 | 325,816 | 604 | 1854 | 182 | 559 |
| 91 | 323,889 | 1285 | 3967 | 401 | 1238 |
| 92 | 323,774 | 1247 | 3851 | 396 | 1223 |
| 93 | 322,640 | 1144 | 3546 | 337 | 1045 |
| 94 | 321,138 | 681 | 2121 | 302 | 940 |
| 95 | 317,136 | 1067 | 3364 | 413 | 1302 |
| 96 | 305,089 | 817 | 2678 | 287 | 941 |
| 97 | 303,709 | 1079 | 3553 | 324 | 1067 |
| 98 | 301,233 | 825 | 2739 | 268 | 890 |
| 99 | 300,597 | 871 | 2898 | 219 | 729 |
| 100 | 297,277 | 1028 | 3458 | 273 | 918 |
| 101 | 291,294 | 1016 | 3488 | 405 | 1390 |
| 102 | 286,612 | 1256 | 4382 | 194 | 677 |
| 103 | 285,967 | 800 | 2798 | 280 | 979 |
| 104 | 282,659 | 1096 | 3877 | 315 | 1114 |
| 105 | 282,316 | 990 | 3507 | 221 | 783 |
| 106 | 279,201 | 760 | 2722 | 260 | 931 |
| 107 | 274,363 | 825 | 3007 | 322 | 1174 |
| 108 | 273,883 | 808 | 2950 | 298 | 1088 |
| 109 | 269,211 | 652 | 2422 | 191 | 709 |
| 110 | 267,671 | 953 | 3560 | 309 | 1154 |
| 111 | 256,930 | 725 | 2822 | 209 | 813 |
| 112 | 253,705 | 627 | 2471 | 215 | 847 |
| 113 | 246,844 | 676 | 2739 | 208 | 843 |
| 114 | 243,291 | 976 | 4012 | 301 | 1237 |
| 115 | 241,074 | 878 | 3642 | 353 | 1464 |
| 116 | 241,053 | 451 | 1871 | 193 | 801 |
| 117 | 240,445 | 942 | 3918 | 267 | 1110 |
| 118 | 237,035 | 584 | 2464 | 189 | 797 |
| 119 | 236,667 | 818 | 3456 | 411 | 1737 |
| 120 | 236,001 | 643 | 2725 | 110 | 466 |
| 121 | 232,661 | 647 | 2781 | 245 | 1053 |
| 122 | 231,588 | 605 | 2612 | 216 | 933 |
| 123 | 227,113 | 665 | 2928 | 293 | 1290 |
| 124 | 225,078 | 796 | 3537 | 271 | 1204 |
| 125 | 222,583 | 643 | 2889 | 323 | 1451 |
| 126 | 221,554 | 418 | 1887 | 141 | 636 |
| 127 | 220,777 | 382 | 1730 | 157 | 711 |
| 128 | 218,452 | 567 | 2596 | 203 | 929 |
| 129 | 216,559 | 576 | 2660 | 130 | 600 |
| 130 | 216,435 | 440 | 2033 | 108 | 499 |
| 131 | 215,987 | 504 | 2333 | 155 | 718 |
| 132 | 214,119 | 491 | 2293 | 115 | 537 |
| 133 | 211,289 | 1461 | 6915 | 457 | 2163 |
| 134 | 211,004 | 691 | 3275 | 225 | 1066 |
| 135 | 210,913 | 563 | 2669 | 242 | 1147 |
| 136 | 210,745 | 451 | 2140 | 107 | 508 |
| 137 | 205,186 | 666 | 3246 | 271 | 1321 |
| 138 | 204,973 | 798 | 3893 | 277 | 1351 |
| 139 | 202,791 | 961 | 4739 | 327 | 1612 |
| 140 | 201,574 | 589 | 2922 | 217 | 1077 |
| 141 | 199,451 | 737 | 3695 | 257 | 1289 |
| 142 | 198,819 | 972 | 4889 | 265 | 1333 |
| 143 | 197,154 | 1416 | 7182 | 397 | 2014 |
| 144 | 195,335 | 410 | 2099 | 129 | 660 |
| 145 | 193,037 | 586 | 3036 | 288 | 1492 |
| 146 | 185,906 | 667 | 3588 | 105 | 565 |
| 147 | 182,655 | 1044 | 5716 | 388 | 2124 |
| 148 | 181,895 | 326 | 1792 | 141 | 775 |
| 149 | 181,884 | 0 | 0 | 0 | 0 |
| 150 | 180,680 | 278 | 1539 | 117 | 648 |
| 151 | 178,533 | 559 | 3131 | 236 | 1322 |
| 152 | 175,847 | 292 | 1661 | 151 | 859 |
| 153 | 174,962 | 437 | 2498 | 147 | 840 |
| 154 | 171,879 | 664 | 3863 | 241 | 1402 |
| 155 | 171,086 | 649 | 3793 | 245 | 1432 |
| 156 | 170,000 | 828 | 4871 | 218 | 1282 |
| 157 | 168,986 | 770 | 4557 | 151 | 894 |
| 158 | 166,708 | 373 | 2237 | 186 | 1116 |
| 159 | 166,185 | 504 | 3033 | 243 | 1462 |
| 160 | 165,042 | 658 | 3987 | 191 | 1157 |
| 161 | 163,805 | 472 | 2881 | 129 | 788 |
| 162 | 161,552 | 373 | 2309 | 176 | 1089 |
| 163 | 161,316 | 429 | 2659 | 145 | 899 |
| 164 | 158,303 | 460 | 2906 | 185 | 1169 |
| 165 | 158,095 | 451 | 2853 | 213 | 1347 |
| 166 | 157,673 | 380 | 2410 | 114 | 723 |
| 167 | 157,458 | 632 | 4014 | 124 | 788 |
| 168 | 152,254 | 379 | 2489 | 185 | 1215 |
| 169 | 151,140 | 713 | 4717 | 183 | 1211 |
| 170 | 149,069 | 285 | 1912 | 111 | 745 |
| 171 | 146,407 | 323 | 2206 | 136 | 929 |
| 172 | 145,668 | 380 | 2609 | 101 | 693 |
| 173 | 142,987 | 277 | 1937 | 133 | 930 |
| 174 | 139,408 | 344 | 2468 | 136 | 976 |
| 175 | 138,178 | 469 | 3394 | 114 | 825 |
| 176 | 136,546 | 351 | 2571 | 143 | 1047 |
| 177 | 135,793 | 379 | 2791 | 123 | 906 |
| 178 | 135,445 | 338 | 2495 | 142 | 1048 |
| 179 | 132,082 | 523 | 3960 | 150 | 1136 |
| 180 | 131,809 | 913 | 6927 | 282 | 2139 |
| 181 | 131,179 | 423 | 3225 | 119 | 907 |
| 182 | 131,079 | 393 | 2998 | 126 | 961 |
| 183 | 130,969 | 448 | 3421 | 114 | 870 |
| 184 | 130,089 | 214 | 1645 | 88 | 676 |
| 185 | 129,738 | 356 | 2744 | 153 | 1179 |
| 186 | 128,813 | 549 | 4262 | 165 | 1281 |
| 187 | 128,787 | 436 | 3385 | 94 | 730 |
| 188 | 128,495 | 540 | 4202 | 170 | 1323 |
| 189 | 124,893 | 337 | 2698 | 167 | 1337 |
| 190 | 123,122 | 258 | 2095 | 60 | 487 |
| 191 | 121,679 | 340 | 2794 | 142 | 1167 |
| 192 | 120,502 | 296 | 2456 | 105 | 871 |
| 193 | 119,084 | 230 | 1931 | 113 | 949 |
| 194 | 118,295 | 281 | 2375 | 78 | 659 |
| 195 | 118,253 | 259 | 2190 | 33 | 279 |
| 196 | 117,697 | 453 | 3849 | 144 | 1223 |
| 197 | 115,102 | 150 | 1303 | 47 | 408 |
| 198 | 113,227 | 274 | 2420 | 98 | 866 |
| 199 | 111,941 | 225 | 2010 | 55 | 491 |
| 200 | 111,299 | 238 | 2138 | 112 | 1006 |
| 201 | 109,818 | 315 | 2868 | 124 | 1129 |
| 202 | 107,917 | 273 | 2530 | 64 | 593 |
| 203 | 107,465 | 391 | 3638 | 161 | 1498 |
| 204 | 132,186 | 271 | 2050 | 31 | 235 |
| 205 | 107,396 | 319 | 2970 | 56 | 521 |
| 206 | 106,748 | 322 | 3016 | 65 | 609 |
| 207 | 106,523 | 166 | 1558 | 40 | 376 |
| 208 | 106,167 | 217 | 2044 | 134 | 1262 |
| 209 | 104,922 | 215 | 2049 | 93 | 886 |
| 210 | 104,207 | 238 | 2284 | 97 | 931 |
| 211 | 103,389 | 364 | 3521 | 161 | 1557 |
| 212 | 101,795 | 202 | 1984 | 76 | 747 |
| 213 | 100,778 | 236 | 2342 | 36 | 357 |
| 214 | 100,166 | 407 | 4063 | 209 | 2087 |
| 215 | 99,468 | 270 | 2714 | 62 | 623 |
| 216 | 99,313 | 230 | 2316 | 86 | 866 |
| 217 | 99,077 | 181 | 1827 | 40 | 404 |
| 218 | 96,772 | 109 | 1126 | 53 | 548 |
| 219 | 96,722 | 322 | 3329 | 139 | 1437 |
| 220 | 95,747 | 384 | 4011 | 145 | 1514 |
| 221 | 95,185 | 277 | 2910 | 100 | 1051 |
| 222 | 94,903 | 274 | 2887 | 80 | 843 |
| 223 | 94,457 | 296 | 3134 | 144 | 1525 |
| 224 | 93,177 | 266 | 2855 | 92 | 987 |
| 225 | 92,908 | 297 | 3197 | 74 | 796 |
| 226 | 92,110 | 268 | 2910 | 113 | 1227 |
| 227 | 90,897 | 311 | 3421 | 113 | 1243 |
| 228 | 89,924 | 208 | 2313 | 56 | 623 |
| 229 | 87,751 | 290 | 3305 | 119 | 1356 |
| 230 | 84,840 | 236 | 2782 | 115 | 1355 |
| 231 | 84,328 | 177 | 2099 | 97 | 1150 |
| 232 | 84,298 | 163 | 1934 | 86 | 1020 |
| 233 | 84,224 | 136 | 1615 | 68 | 807 |
| 234 | 83,871 | 320 | 3815 | 141 | 1681 |
| 235 | 82,532 | 236 | 2859 | 92 | 1115 |
| 236 | 82,019 | 251 | 3060 | 49 | 597 |
| 237 | 81,786 | 227 | 2776 | 105 | 1284 |
| 238 | 81,587 | 349 | 4278 | 113 | 1385 |
| 239 | 79,946 | 207 | 2589 | 106 | 1326 |
| 240 | 79,583 | 146 | 1835 | 53 | 666 |
| 241 | 79,542 | 225 | 2829 | 78 | 981 |
| 242 | 79,184 | 231 | 2917 | 88 | 1111 |
| 243 | 77,936 | 109 | 1399 | 77 | 988 |
| 244 | 77,214 | 171 | 2215 | 57 | 738 |
| 245 | 76,672 | 277 | 3613 | 37 | 483 |
| 246 | 75,692 | 185 | 2444 | 111 | 1466 |
| 247 | 75,203 | 92 | 1223 | 28 | 372 |
| 248 | 75,031 | 168 | 2239 | 81 | 1080 |
| 249 | 73,924 | 181 | 2448 | 80 | 1082 |
| 250 | 73,606 | 105 | 1427 | 70 | 951 |
| 251 | 73,189 | 101 | 1380 | 47 | 642 |
| 252 | 72,845 | 234 | 3212 | 82 | 1126 |
| 253 | 72,737 | 380 | 5224 | 110 | 1512 |
| 254 | 72,504 | 69 | 952 | 54 | 745 |
| 255 | 71,906 | 274 | 3811 | 54 | 751 |
| 256 | 71,495 | 97 | 1357 | 38 | 532 |
| 257 | 71,029 | 150 | 2112 | 58 | 817 |
| 258 | 70,688 | 198 | 2801 | 73 | 1033 |
| 259 | 69,413 | 99 | 1426 | 29 | 418 |
| 260 | 69,283 | 79 | 1140 | 43 | 621 |
| 261 | 67,919 | 73 | 1075 | 42 | 618 |
| 262 | 67,620 | 141 | 2085 | 23 | 340 |
| 263 | 67,127 | 118 | 1758 | 76 | 1132 |
| 264 | 66,715 | 62 | 929 | 24 | 360 |
| 265 | 66,608 | 99 | 1486 | 61 | 916 |
| 266 | 66,504 | 277 | 4165 | 127 | 1910 |
| 267 | 65,977 | 222 | 3365 | 37 | 561 |
| 268 | 65,579 | 250 | 3812 | 102 | 1555 |
| 269 | 63,505 | 2 | 31 | 1 | 16 |
| 270 | 63,451 | 121 | 1907 | 58 | 914 |
| 271 | 62,377 | 83 | 1331 | 12 | 192 |
| 272 | 60,393 | 218 | 3610 | 69 | 1143 |
| 273 | 59,609 | 148 | 2483 | 19 | 319 |
| 274 | 58,083 | 327 | 5630 | 70 | 1205 |
| 275 | 57,094 | 70 | 1226 | 27 | 473 |
| 276 | 56,838 | 213 | 3747 | 101 | 1777 |
| 277 | 56,637 | 349 | 6162 | 129 | 2278 |
| 278 | 56,549 | 136 | 2405 | 37 | 654 |
| 279 | 55,306 | 134 | 2423 | 34 | 615 |
| 280 | 55,283 | 207 | 3744 | 16 | 289 |
| 281 | 55,206 | 62 | 1123 | 12 | 217 |
| 282 | 54,729 | 187 | 3417 | 77 | 1407 |
| 283 | 54,588 | 154 | 2821 | 47 | 861 |
| 284 | 54,468 | 183 | 3360 | 28 | 514 |
| 285 | 53,790 | 53 | 985 | 43 | 799 |
| 286 | 53,437 | 67 | 1254 | 27 | 505 |
| 287 | 52,843 | 181 | 3425 | 111 | 2101 |
| 288 | 52,768 | 248 | 4700 | 68 | 1289 |
| 289 | 52,490 | 115 | 2191 | 27 | 514 |
| 290 | 51,995 | 138 | 2654 | 24 | 462 |
| 291 | 51,488 | 144 | 2797 | 57 | 1107 |
| 292 | 51,449 | 133 | 2585 | 54 | 1050 |
| 293 | 51,276 | 126 | 2457 | 52 | 1014 |
| 294 | 51,268 | 89 | 1736 | 44 | 858 |
| 295 | 50,457 | 114 | 2259 | 22 | 436 |
| 296 | 50,036 | 137 | 2738 | 32 | 640 |
| 297 | 49,729 | 128 | 2574 | 46 | 925 |
| 298 | 49,541 | 184 | 3714 | 46 | 929 |
| 299 | 49,354 | 101 | 2046 | 54 | 1094 |
| 300 | 48,946 | 123 | 2513 | 30 | 613 |
| 301 | 48,836 | 85 | 1741 | 14 | 287 |
| 302 | 48,572 | 171 | 3521 | 18 | 371 |
| 303 | 48,164 | 55 | 1142 | 24 | 498 |
| 304 | 48,005 | 95 | 1979 | 21 | 437 |
| 305 | 48,005 | 73 | 1521 | 48 | 1000 |
| 306 | 46,186 | 84 | 1819 | 51 | 1104 |
| 307 | 46,063 | 75 | 1628 | 56 | 1216 |
| 308 | 45,799 | 66 | 1441 | 31 | 677 |
| 309 | 45,609 | 166 | 3640 | 45 | 987 |
| 310 | 45,554 | 15 | 329 | 3 | 66 |
| 311 | 44,841 | 184 | 4103 | 39 | 870 |
| 312 | 43,972 | 147 | 3343 | 76 | 1728 |
| 313 | 43,186 | 111 | 2570 | 54 | 1250 |
| 314 | 43,179 | 37 | 857 | 15 | 347 |
| 315 | 40,589 | 71 | 1749 | 22 | 542 |
| 316 | 40,395 | 90 | 2228 | 44 | 1089 |
| 317 | 38,821 | 7 | 180 | 1 | 26 |
| 318 | 38,277 | 115 | 3004 | 17 | 444 |
| 319 | 38,211 | 96 | 2512 | 58 | 1518 |
| 320 | 37,568 | 196 | 5217 | 78 | 2076 |
| 321 | 37,561 | 42 | 1118 | 27 | 719 |
| 322 | 37,548 | 92 | 2450 | 19 | 506 |
| 323 | 36,446 | 120 | 3293 | 23 | 631 |
| 324 | 36,258 | 43 | 1186 | 13 | 359 |
| 325 | 36,166 | 31 | 857 | 17 | 470 |
| 326 | 35,822 | 43 | 1200 | 14 | 391 |
| 327 | 35,366 | 140 | 3959 | 74 | 2092 |
| 328 | 35,081 | 78 | 2223 | 15 | 428 |
| 329 | 35,052 | 112 | 3195 | 20 | 571 |
| 330 | 34,908 | 161 | 4612 | 23 | 659 |
| 331 | 34,292 | 111 | 3237 | 27 | 787 |
| 332 | 33,730 | 84 | 2490 | 12 | 356 |
| 333 | 32,925 | 21 | 638 | 14 | 425 |
| 334 | 32,495 | 71 | 2185 | 19 | 585 |
| 335 | 32,365 | 118 | 3646 | 7 | 216 |
| 336 | 32,254 | 26 | 806 | 20 | 620 |
| 337 | 31,271 | 85 | 2718 | 33 | 1055 |
| 338 | 30,617 | 23 | 751 | 14 | 457 |
| 339 | 30,247 | 16 | 529 | 10 | 331 |
| 340 | 29,918 | 25 | 836 | 16 | 535 |
| 341 | 29,858 | 50 | 1675 | 7 | 234 |
| 342 | 29,184 | 11 | 377 | 1 | 34 |
| 343 | 28,908 | 0 | 0 | 0 | 0 |
| 344 | 28,404 | 46 | 1619 | 15 | 528 |
| 345 | 27,855 | 38 | 1364 | 7 | 251 |
| 346 | 27,495 | 24 | 873 | 15 | 546 |
| 347 | 26,488 | 30 | 1133 | 21 | 793 |
| 348 | 26,340 | 107 | 4062 | 24 | 911 |
| 349 | 26,252 | 102 | 3885 | 12 | 457 |
| 350 | 25,789 | 109 | 4227 | 29 | 1125 |
| 351 | 25,780 | 75 | 2909 | 3 | 116 |
| 352 | 25,728 | 95 | 3692 | 4 | 155 |
| 353 | 25,469 | 52 | 2042 | 41 | 1610 |
| 354 | 25,118 | 32 | 1274 | 9 | 358 |
| 355 | 25,007 | 31 | 1240 | 19 | 760 |
| 356 | 24,974 | 40 | 1602 | 3 | 120 |
| 357 | 24,825 | 27 | 1088 | 10 | 403 |
| 358 | 24,641 | 39 | 1583 | 26 | 1055 |
| 359 | 24,631 | 108 | 4385 | 19 | 771 |
| 360 | 24,110 | 25 | 1037 | 10 | 415 |
| 361 | 23,808 | 28 | 1176 | 39 | 1638 |
| 362 | 23,454 | 23 | 981 | 16 | 682 |
| 363 | 23,352 | 43 | 1841 | 8 | 343 |
| 364 | 23,216 | 37 | 1594 | 15 | 646 |
| 365 | 23,198 | 20 | 862 | 6 | 259 |
| 366 | 22,940 | 108 | 4708 | 28 | 1221 |
| 367 | 22,928 | 54 | 2355 | 18 | 785 |
| 368 | 22,775 | 4 | 176 | 4 | 176 |
| 369 | 22,636 | 10 | 442 | 3 | 133 |
| 370 | 22,310 | 85 | 3810 | 161 | 7216 |
| 371 | 22,305 | 30 | 1345 | 8 | 359 |
| 372 | 22,225 | 86 | 3870 | 27 | 1215 |
| 373 | 21,927 | 107 | 4880 | 55 | 2508 |
| 374 | 21,778 | 57 | 2617 | 35 | 1607 |
| 375 | 21,723 | 40 | 1841 | 2 | 92 |
| 376 | 21,585 | 53 | 2455 | 18 | 834 |
| 377 | 21,255 | 33 | 1553 | 15 | 706 |
| 378 | 20,976 | 18 | 858 | 5 | 238 |
| 379 | 20,505 | 32 | 1561 | 13 | 634 |
| 380 | 20,261 | 70 | 3455 | 16 | 790 |
| 381 | 20,043 | 10 | 499 | 3 | 150 |

**Table S4** Statistics of Indels of each mutant isolate in terms of insertions, deletions, and different lengths

| Mutant | Indels | Insertion | | % | Deletion | % | 1bp Indels | % | 2bp  Indels | % | 3bp  Indels | % | ≤ 3bp (%) | Longest Indels |
| --- | --- | --- | --- | --- | --- | --- | --- | --- | --- | --- | --- | --- | --- | --- |
| M11-Yr36-1 | 20,705 | 11,832 | 57.15 | | 8,873 | 42.85 | 8447 | 40.8 | 4239 | 20.47 | 2148 | 10.37 | 71.64 | -185 |
| M11-YrTr1 | 19,102 | 11,125 | 58.24 | | 7,977 | 41.76 | 7981 | 41.78 | 3843 | 20.12 | 1927 | 10.09 | 71.99 | -203 |
| M11-Yr21 | 17,671 | 10,400 | 58.85 | | 7,271 | 41.15 | 7521 | 42.56 | 3584 | 20.28 | 1724 | 9.76 | 72.6 | 224 |
| M11-YrSP-2 | 17,654 | 11,225 | 63.58 | | 6,429 | 36.42 | 8774 | 49.7 | 3414 | 19.34 | 1633 | 9.25 | 78.29 | 217 |
| M11-Yr9-2 | 17,424 | 10,166 | 58.34 | | 7,258 | 41.66 | 7397 | 42.45 | 3576 | 20.52 | 1721 | 9.88 | 72.85 | 229 |
| M11-YrSP-1 | 17,187 | 11,007 | 64.04 | | 6,180 | 35.96 | 8624 | 50.18 | 3309 | 19.25 | 1566 | 9.11 | 78.54 | -203 |
| M11-YrExp2 | 17,024 | 10,924 | 64.17 | | 6,100 | 35.83 | 8499 | 49.92 | 3303 | 19.4 | 1548 | 9.09 | 78.42 | 217 |
| M11-Yr76-2 | 16,591 | 10,792 | 65.05 | | 5,799 | 34.95 | 8344 | 50.29 | 3195 | 19.26 | 1466 | 8.84 | 78.39 | 217 |
| M11-Paha | 15,814 | 10,425 | 65.92 | | 5,389 | 34.08 | 8124 | 51.37 | 2955 | 18.69 | 1408 | 8.9 | 78.96 | -190 |
| M11-Yr36-2 | 15,768 | 10,407 | 66 | | 5,361 | 34 | 8128 | 51.55 | 2969 | 18.83 | 1385 | 8.78 | 79.16 | -190 |
| M11-Yr2-2 | 15,597 | 10,377 | 66.53 | | 5,220 | 33.47 | 8135 | 52.16 | 2934 | 18.81 | 1318 | 8.45 | 79.42 | -185 |
| M11-Yr2-1 | 15,546 | 10,282 | 66.14 | | 5,264 | 33.86 | 8111 | 52.17 | 2899 | 18.65 | 1370 | 8.81 | 79.63 | 217 |
| M11-Yr24-1 | 14,645 | 8,937 | 61.02 | | 5,708 | 38.98 | 6599 | 45.06 | 2840 | 19.39 | 1341 | 9.16 | 73.61 | **-245** |
| M11-Yr6 | 14,480 | 8,666 | 59.85 | | 5,814 | 40.15 | 6147 | 42.45 | 2897 | 20.01 | 1398 | 9.65 | 72.11 | 242 |
| M11-Yr1-3 | 13,663 | 9,016 | 65.99 | | 4,647 | 34.01 | 6943 | 50.82 | 2611 | 19.11 | 1185 | 8.67 | 78.6 | 217 |
| M11-Yr9-4 | 13,594 | 9,081 | 66.8 | | 4,513 | 33.2 | 7087 | 52.13 | 2516 | 18.51 | 1109 | 8.16 | 78.8 | -203 |
| M11-Yr9-1 | 13,497 | 8,122 | 60.18 | | 5,375 | 39.82 | 5830 | 43.19 | 2694 | 19.96 | 1245 | 9.22 | 72.38 | -236 |
| M11-Yr1-2 | 13,475 | 9,124 | 67.71 | | 4,351 | 32.29 | 5368 | 39.84 | 1602 | 11.89 | 676 | 5.02 | 56.74 | -185 |
| M11-Fielder | 13,402 | 8,172 | 60.98 | | 5,230 | 39.02 | 5809 | 43.34 | 2676 | 19.97 | 1215 | 9.07 | 72.38 | **273** |
| M11-Yr10 | 12,015 | 7,455 | 62.05 | | 4,560 | 37.95 | 5351 | 44.54 | 2328 | 19.38 | 1099 | 9.15 | 73.06 | 229 |
| M11-Yr17 | 11,600 | 7,412 | 63.9 | | 4,188 | 36.1 | 5616 | 48.41 | 2202 | 18.98 | 1026 | 8.84 | 76.24 | -185 |
| M11-YrA+ | 11,098 | 7,038 | 63.42 | | 4,060 | 36.58 | 5155 | 46.45 | 2128 | 19.17 | 957 | 8.62 | 74.25 | -236 |
| M11-Yr39 | 9,919 | 6,416 | 64.68 | | 3,503 | 35.32 | 4839 | 48.79 | 1901 | 19.17 | 817 | 8.24 | 76.19 | -203 |
| M11-Yr76-1 | 8,897 | 5,555 | 62.44 | | 3,342 | 37.56 | 3848 | 43.25 | 1673 | 18.8 | 827 | 9.3 | 71.35 | 174 |
| M11-Yr76-3 | 7,236 | 4,830 | 66.75 | | 2,406 | 33.25 | 3624 | 50.08 | 1275 | 17.62 | 603 | 8.33 | 76.04 | -203 |
| M11-Yr43 | 5,951 | 3,729 | 62.66 | | 2,222 | 37.34 | 2676 | 44.97 | 1085 | 18.23 | 505 | 8.49 | 71.69 | 217 |
| M11-Yr44 | 4,454 | 2,945 | 66.12 | | 1,509 | 33.88 | 1951 | 43.8 | 823 | 18.48 | 368 | 8.26 | 70.54 | 206 |
| M11-Yr8 | 4,367 | 2,846 | 65.17 | | 1,521 | 34.83 | 2044 | 46.81 | 583 | 13.35 | 248 | 5.68 | 65.83 | 263 |
| M11-Yr31 | 4,188 | 2,826 | 67.48 | | 1,362 | 32.52 | 1909 | 45.58 | 755 | 18.03 | 315 | 7.52 | 71.13 | -177 |
| M11-Yr1-1 | 4,005 | 2,764 | 69.01 | | 1,241 | 30.99 | 2102 | 52.48 | 682 | 17.03 | 275 | 6.87 | 76.38 | 177 |
| **Average** | **12,886** | **8,130** | **63.67** | | **4,756** | **36.33** | **6,033** | **46.90** | **2,450** | **18.69** | **1,147** | **8.7** | **74.24** | **/** |

**Table S5** Number of SNPs, Indels, insertions and deletions of each mutant isolate identified from 11-281 haplotigs

| Mutant | No. of | No. of | No. of | No. of |
| --- | --- | --- | --- | --- |
| isolates | SNP | Indel | insertion | deletion |
| M11-Yr21 | 60,581 | 44,895 | 33,144 | 11,751 |
| M11-YrExp2 | 57,986 | 40,256 | 29,933 | 10,323 |
| M11-Yr9-4 | 54,476 | 39,801 | 29,497 | 10,304 |
| M11-Yr31 | 52,286 | 38,160 | 28,203 | 9,957 |
| M11-Yr43 | 49,822 | 35,558 | 26,365 | 9,193 |
| M11-Yr1-2 | 45,984 | 29,619 | 22,151 | 7,468 |
| M11-Paha | 42,753 | 16,730 | 12,247 | 4,483 |
| M11-Yr2-2 | 35,023 | 24,072 | 17,897 | 6,175 |
| M11-Fielder | 31,104 | 19,434 | 14,291 | 5,143 |
| M11-Yr76-2 | 30,485 | 20,472 | 15,178 | 5,294 |
| M11-Yr39 | 30,280 | 20,572 | 15,128 | 5,444 |
| M11-Yr8 | 24,740 | 9,384 | 7,072 | 2,312 |
| M11-YrTr1 | 24,588 | 14,376 | 10,531 | 3,845 |
| M11-YrSP-1 | 24,263 | 15,797 | 11,660 | 4,137 |
| M11-Yr76-1 | 20,375 | 12,553 | 9,247 | 3,306 |
| M11-Yr2-1 | 17,854 | 10,242 | 7,657 | 2,585 |
| M11-Yr1-3 | 17,403 | 10,724 | 7,990 | 2,734 |
| M11-Yr6 | 17,114 | 10,117 | 7,456 | 2,661 |
| M11-Yr1-1 | 15,254 | 7,544 | 5,544 | 2,000 |
| M11-YrA+ | 14,622 | 8,853 | 6,597 | 2,256 |
| M11-Yr9-1 | 14,304 | 8,319 | 6,163 | 2,156 |
| M11-Yr36-1 | 13,241 | 7,607 | 5,548 | 2,059 |
| M11-YrSP-2 | 11,962 | 6,788 | 5,106 | 1,682 |
| M11-Yr36-2 | 10,281 | 4,999 | 3,767 | 1,232 |
| M11-Yr76-3 | 7,252 | 3,773 | 2,794 | 979 |
| M11-Yr10 | 6,081 | 1,152 | 385 | 767 |
| M11-Yr9-2 | 4,729 | 795 | 283 | 512 |
| M11-Yr24-1 | 3,327 | 518 | 194 | 324 |
| M11-Yr44 | 3,129 | 586 | 209 | 377 |
| M11-Yr17 | 2,566 | 357 | 136 | 221 |
| **Average** | **24,796** | **15,468** | **11,412** | **4,056** |

**Table S6** Genes highly associated to avirulence with *P*-value ≤ 0.001

| Variant | *Avr* gene | Variant type | *P*-value | Position |
| --- | --- | --- | --- | --- |
| PS_11-281_00002262 | *AvYr8* | Missense variant | 1.25E-04 | Scaffold11: 288939 - 291555 |
| PS_11-281_00004920 | *AvYr27* | Missense variant | 1.56E-04 | Scaffold41: 217940 - 219609 |
| PS_11-281_00014472 | *AvYr7* | Missense variant; inframe insertion | 2.02E-04 | Scaffold160: 33302 - 35765 |
| PS_11-281_00015954 | *AvYrSP* | Missense variant | 2.58E-04 | Scaffold217: 37802 - 38653 |
| PS_11-281_00005142 | *AvYr27* | Missense variant | 3.75E-04 | Scaffold33: 25465 - 29223 |
| PS_11-281_00003874 | *AvYr27* | Missense variant | 3.75E-04 | Scaffold16: 324798 - 329659 |
| PS_11-281_00011302 | *AvYrSP* | Frameshift variant | 4.11E-04 | Scaffold93: 232228 - 239823 |
| PS_11-281_haploid_00011106 | *AvYr6* | Missense variant | 4.90E-04 | Haplotig_scaffold545: 6958 - 10612 |
| PS_11-281_00005476 | *AvYr8* | Missense variant | 5.40E-04 | Scaffold22: 307445 - 308876 |
| PS_11-281_00003048 | *AvYr8* | Missense variant | 5.83E-04 | Scaffold19: 521417 - 536500 |
| PS_11-281_00000659 | *AvYrSP* | Missense variant; stop gained | 6.53E-04 | Scaffold1: 340095 - 340987 |
| PS_11-281_00016080 | *AvYr8* | Missense variant; stop gained;  frameshift variant | 7.64E-04 | Scaffold253: 9156 - 14394 |
| PS_11-281_00009555 | *AvYr9* | Missense variant | 8.69E-04 | Scaffold69: 162374 - 164146 |
| PS_11-281_00000903 | *AvYr9* | Missense variant;  inframe insertion | 8.69E-04 | Scaffold17: 278418 - 282803 |
| PS_11-281_haploid_00009111 | *AvYrSP* | Missense variant; frameshift variant | 8.74E-04 | Haplotig_scaffold321: 16655 - 20691 |
| PS_11-281_haploid_00002745 | *AVYr24; AvYr32* | Frameshift variant; start lost | 8.87E-04;  6.17E-04 | Haplotig_scaffold43: 10767 - 14043 |
| PS_11-281_00014479 | *AvYr27* | Stop gained; frameshift variant | 9.26E-04 | Scaffold160: 78364 - 79008 |
