# Supplementary material for: Whole-genome sequencing of Puccinia striiformis f. sp. tritici mutant isolates identifies avirulence gene candidates
Source: BMC Genomics. 2020 Mar 20;21:247. doi: 10.1186/s12864-020-6677-y (PMC7085141; doi:10.1186/s12864-020-6677-y)
Supplement: Supplementary file 2 — Additional file 2: Figure S1. Phylogenetic trees of Puccinia striiformis f. sp. tritici mutant isolates based on the EMS-induced SNPs. Figure S2. Types and frequencies of SNP effects detected from the reference genome haplotigs. A: The number and percentage of all EMS-induced SNPs for each type of effects. B: The types and percentages of deleterious SNP effects. Figure S3. Types and frequencies of Indel effects detected from the reference genome haplotigs. A: The number and percentages of all EMS-induced Indels for each type of effects. B: The types and percentages of deleterious Indel effects. Figure S4. Phylogenetic tree of the progenitor isolate 11–281 of Puccinia striiformis f. sp. tritici (Pst) together with other 12 fungal isolates. The phylogenetic tree was developed from 13 protein sequences using a hill-climbing algorithm with the GTRGAMMA model. 13 fungi isolates are Verticillium dahlia 12,008, Fusarium oxysporum f. sp. cepae FoC_Fus2, Fusarium graminearum ITEM_124, Blumeria graminis f. sp. hordei RACE1, Botrytis cinerea B05.10, Melampsora larici-populina 98AG31, Puccinia striiformis f. sp. tritici 104E137A-, 93–210, 11–281, Puccinia striiformis f. sp. hordei 93TX-2, Puccinia coronata f. sp. avenae 12SD80, Puccinia graminis f. sp. tritici CRL75–36–700-3 and Puccinia triticina BBBD Race 1. The numbers indicate the distances between nodes. Figure S5. Characterization of Puccinia striiformis f. sp. tritici Avr effector candidates in the Upset plot. A: 48 effector candidates of secreted protein (SP) genes. B: 14 effector candidates from non-SP genes. Figure S6. Percentages (%) of the subcellular localization sites of effector candidates of Puccinia striiformis f. sp. tritici in the fungal cells. A: Genes identified from secreted protein (SP) genes. B: Genes identified from non-SP genes. C: All 62 genes associated to avirulence genes. Figure S7. Percentages (%) of the subcellular localization sites of effector candidates of Puccinia striiformis f. sp. tritici i [file 12864_2020_6677_MOESM2_ESM.pptx]

## Slide 1
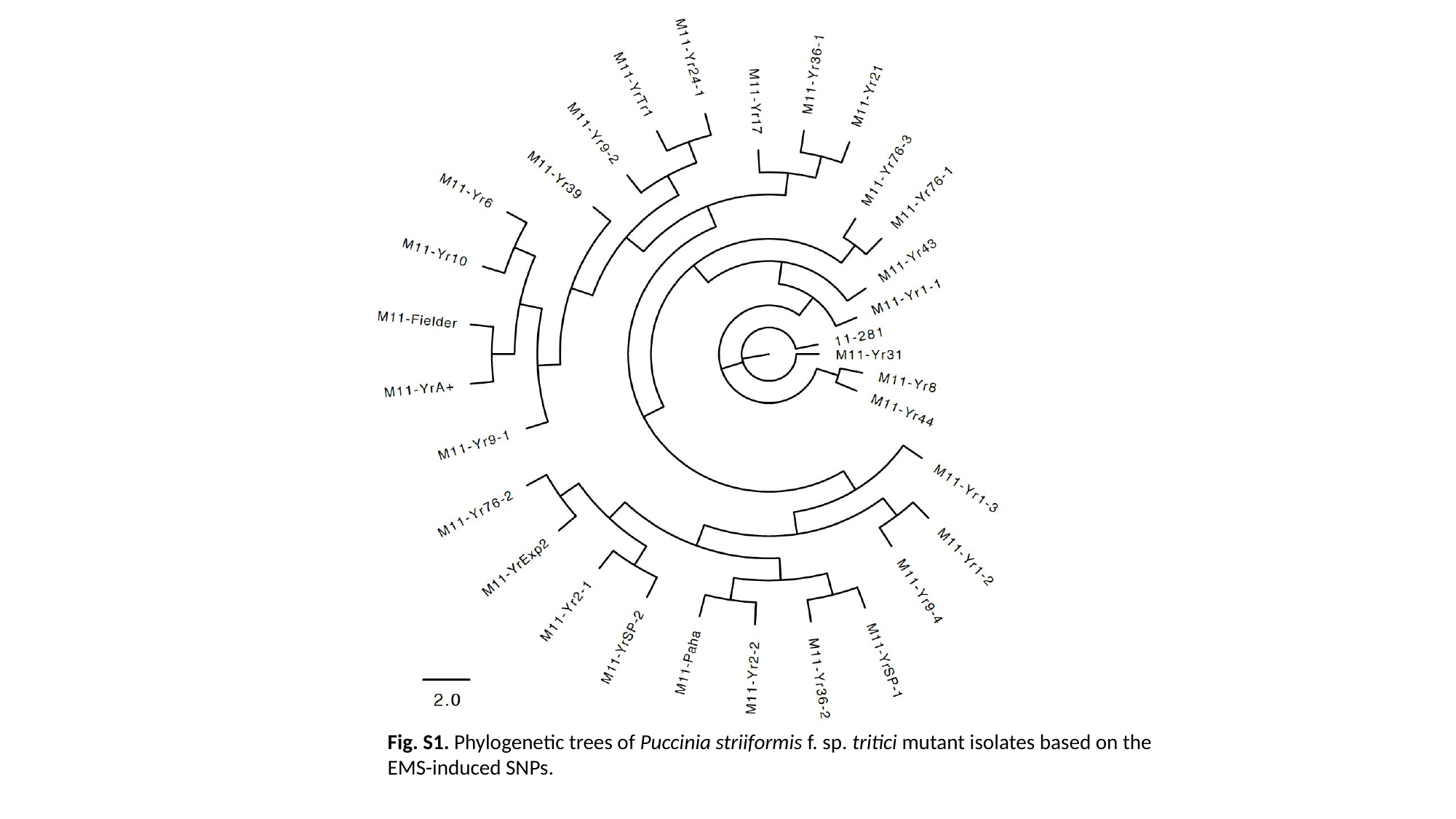

Fig. S1. Phylogenetic trees of Puccinia striiformis f. sp. tritici mutant isolates based on the EMS-induced SNPs.

## Slide 2
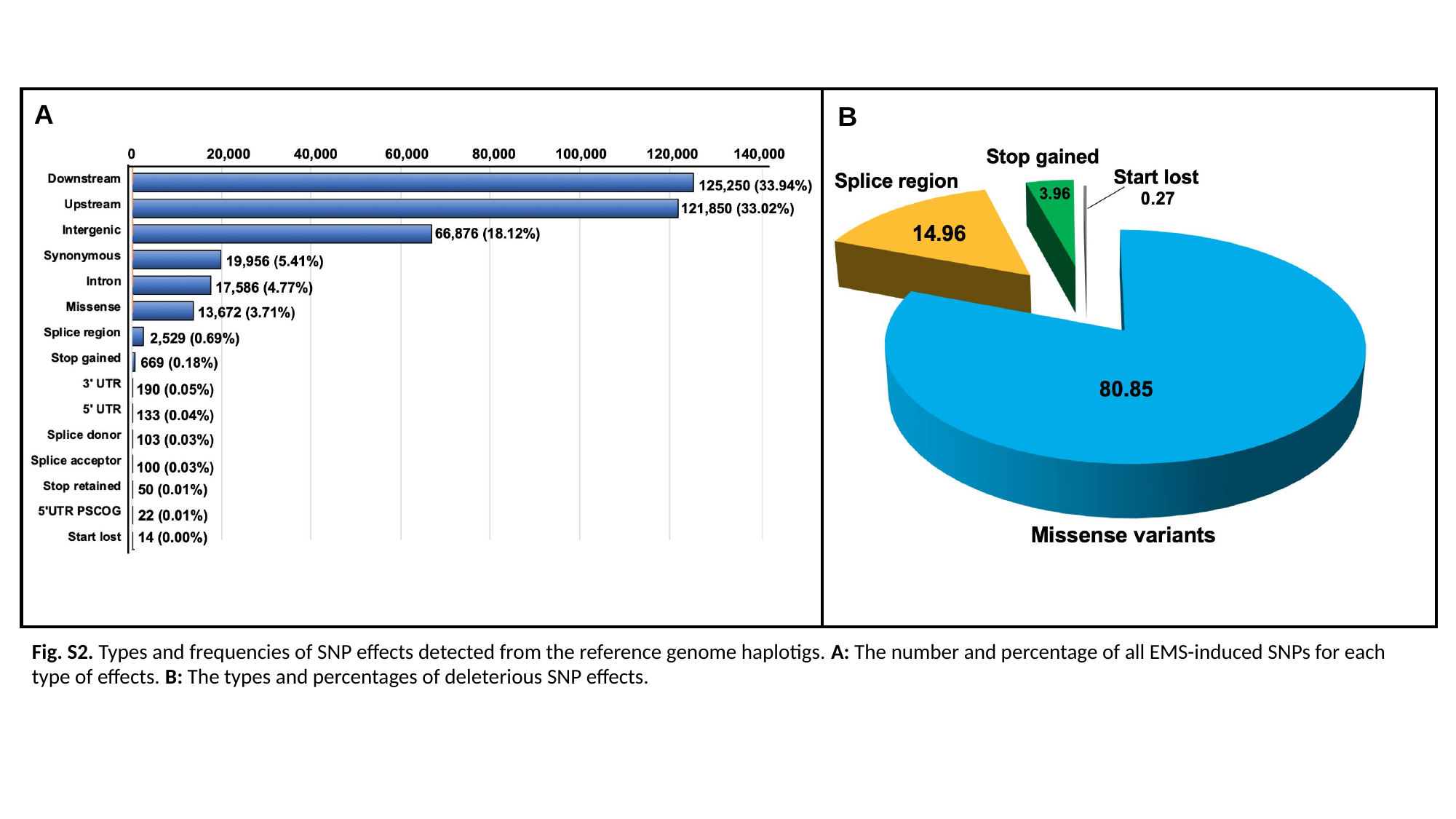

A
B
Fig. S2. Types and frequencies of SNP effects detected from the reference genome haplotigs. A: The number and percentage of all EMS-induced SNPs for each type of effects. B: The types and percentages of deleterious SNP effects.

## Slide 3
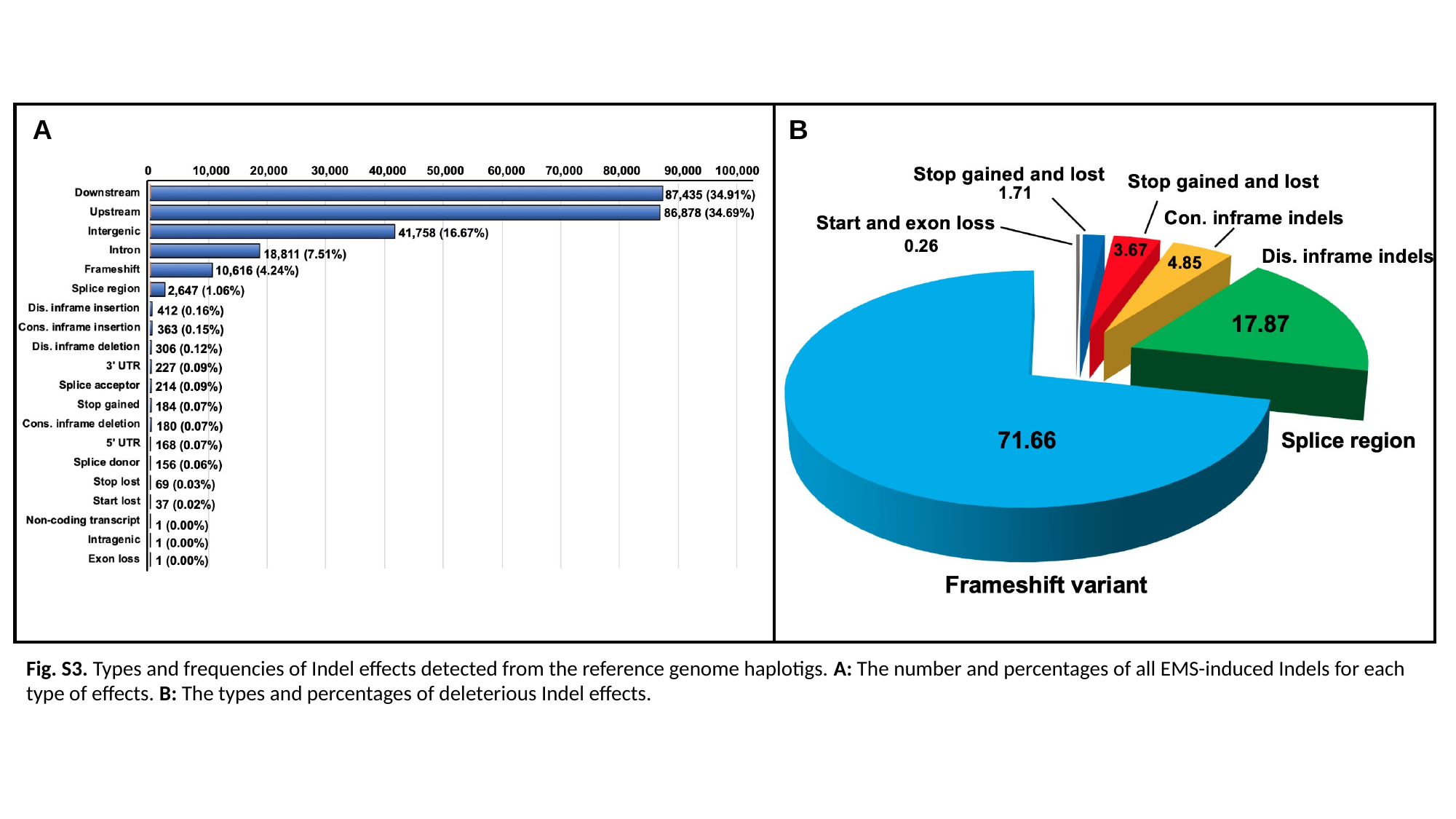

A
B
Fig. S3. Types and frequencies of Indel effects detected from the reference genome haplotigs. A: The number and percentages of all EMS-induced Indels for each type of effects. B: The types and percentages of deleterious Indel effects.

## Slide 4
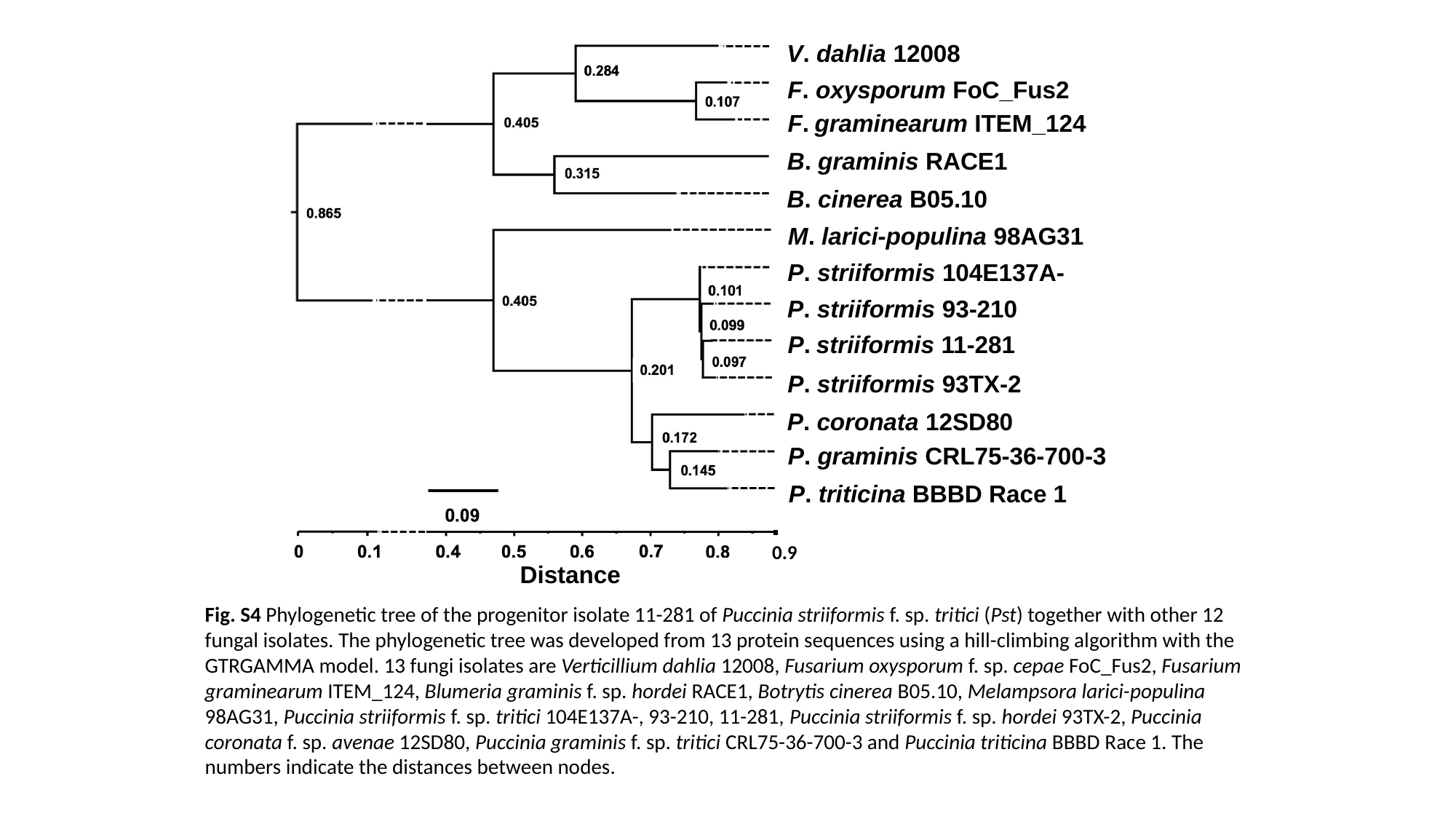

V. dahlia 12008
F. oxysporum FoC_Fus2
F. graminearum ITEM_124
B. graminis RACE1
B. cinerea B05.10
M. larici-populina 98AG31
P. striiformis 104E137A-
P. striiformis 93-210
P. striiformis 93TX-2
P. coronata 12SD80
P. graminis CRL75-36-700-3
P. triticina BBBD Race 1
0.9
Distance
P. striiformis 11-281
Fig. S4 Phylogenetic tree of the progenitor isolate 11-281 of Puccinia striiformis f. sp. tritici (Pst) together with other 12 fungal isolates. The phylogenetic tree was developed from 13 protein sequences using a hill-climbing algorithm with the GTRGAMMA model. 13 fungi isolates are Verticillium dahlia 12008, Fusarium oxysporum f. sp. cepae FoC_Fus2, Fusarium graminearum ITEM_124, Blumeria graminis f. sp. hordei RACE1, Botrytis cinerea B05.10, Melampsora larici-populina 98AG31, Puccinia striiformis f. sp. tritici 104E137A-, 93-210, 11-281, Puccinia striiformis f. sp. hordei 93TX-2, Puccinia coronata f. sp. avenae 12SD80, Puccinia graminis f. sp. tritici CRL75-36-700-3 and Puccinia triticina BBBD Race 1. The numbers indicate the distances between nodes.

## Slide 5
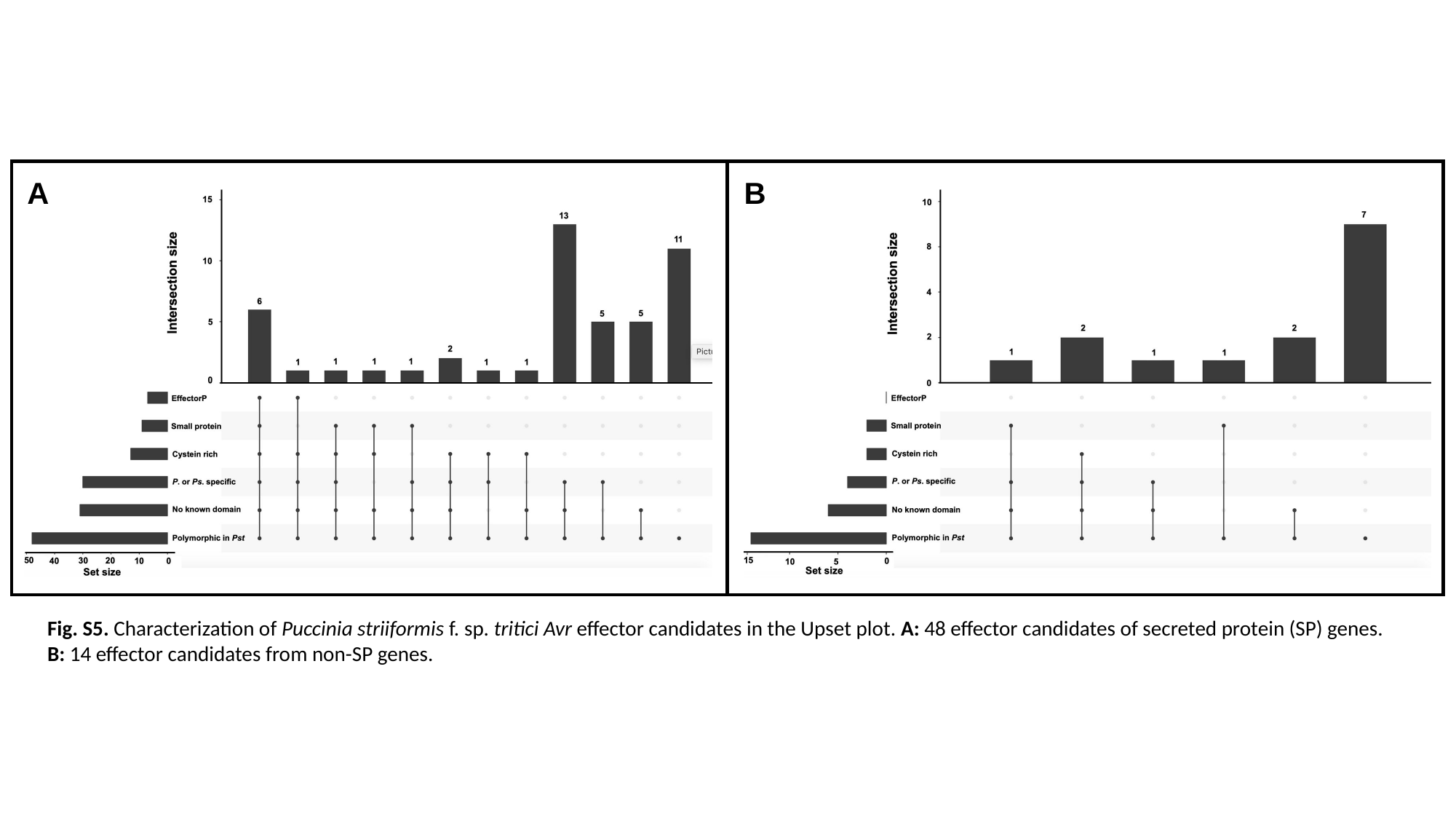

A
B
Fig. S5. Characterization of Puccinia striiformis f. sp. tritici Avr effector candidates in the Upset plot. A: 48 effector candidates of secreted protein (SP) genes. B: 14 effector candidates from non-SP genes.

## Slide 6
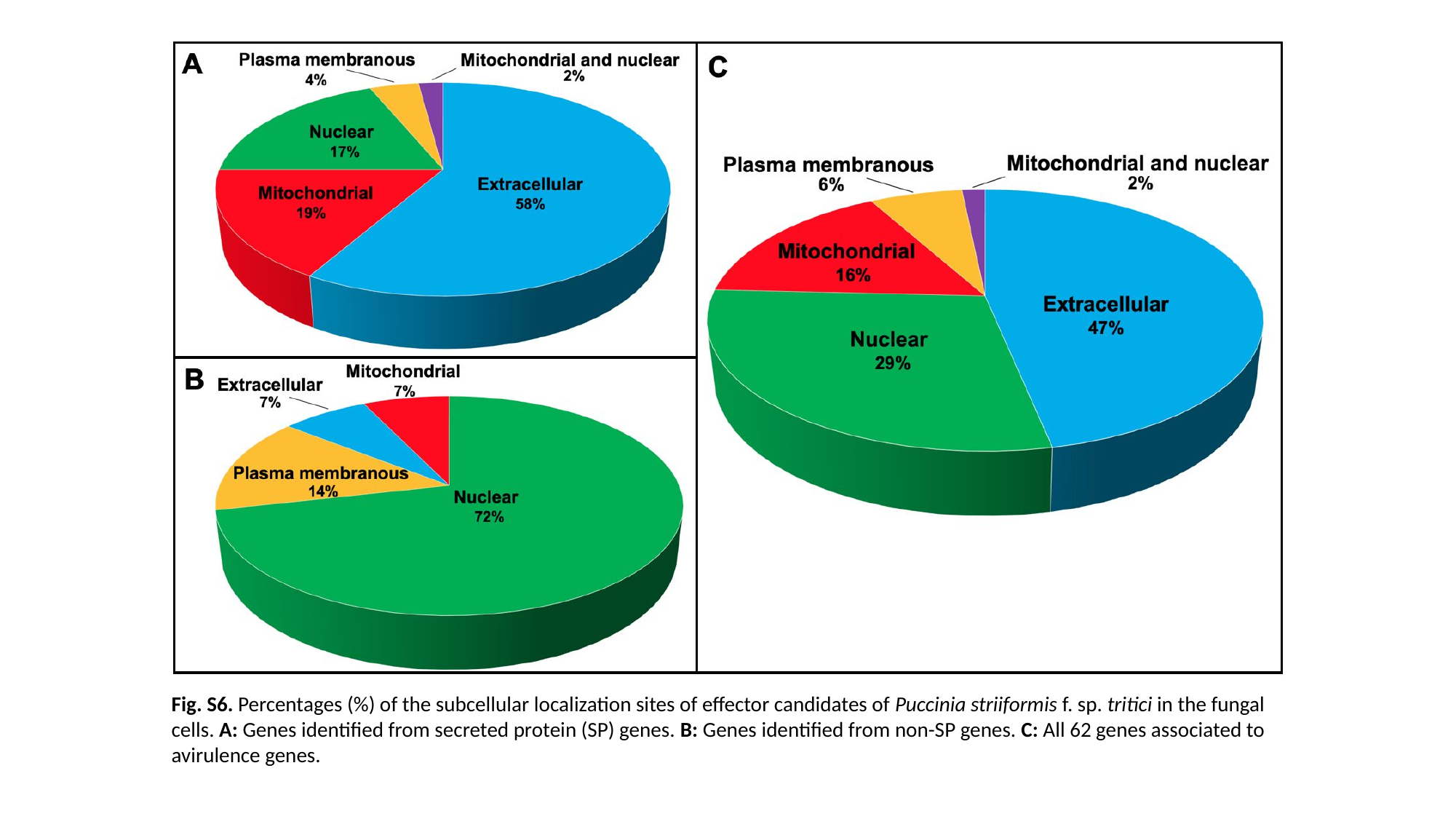

Fig. S6. Percentages (%) of the subcellular localization sites of effector candidates of Puccinia striiformis f. sp. tritici in the fungal cells. A: Genes identified from secreted protein (SP) genes. B: Genes identified from non-SP genes. C: All 62 genes associated to avirulence genes.

## Slide 7
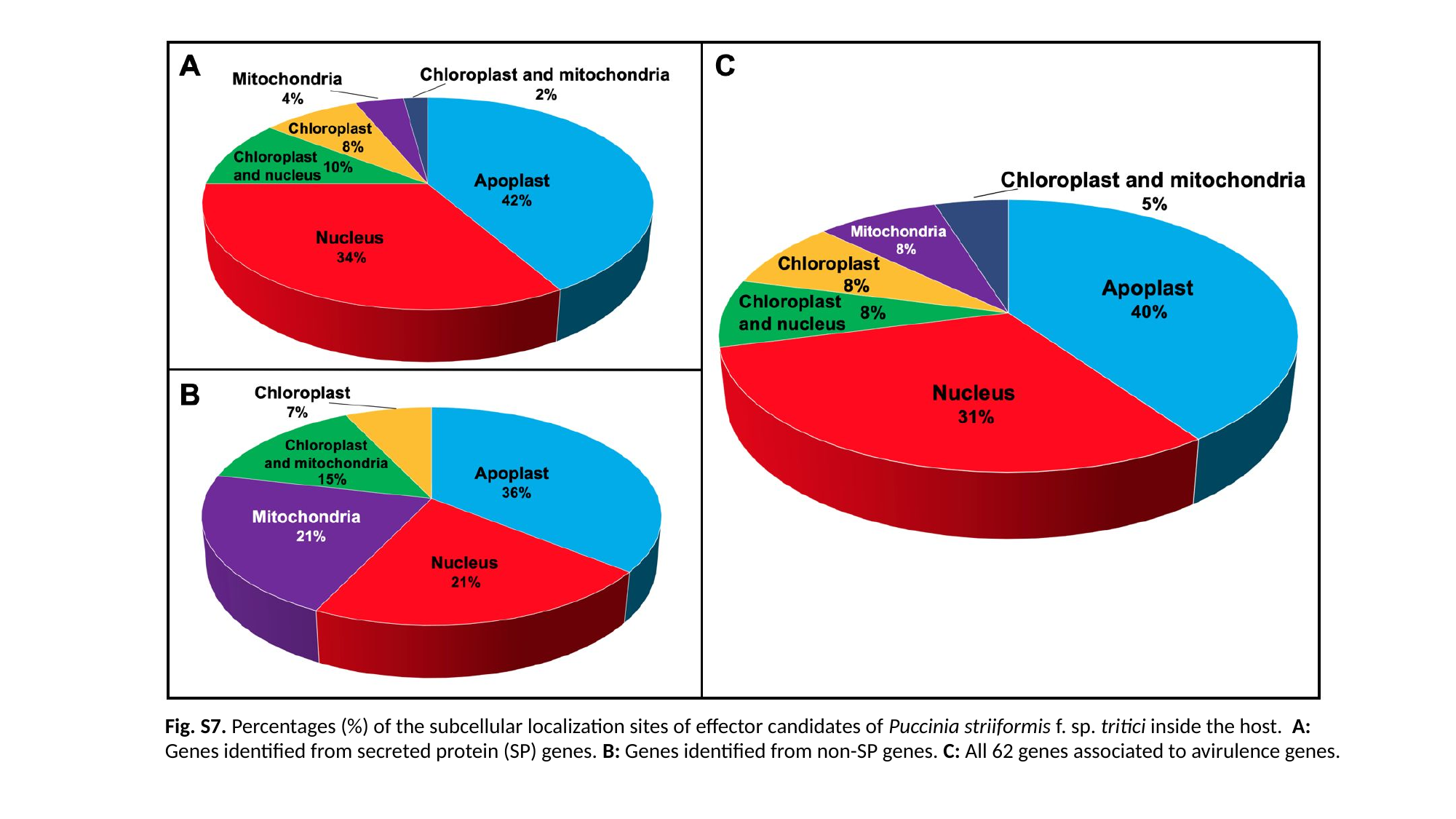

Fig. S7. Percentages (%) of the subcellular localization sites of effector candidates of Puccinia striiformis f. sp. tritici inside the host. A: Genes identified from secreted protein (SP) genes. B: Genes identified from non-SP genes. C: All 62 genes associated to avirulence genes.
